# Single-cell dissection of chronic lung allograft dysfunction reveals convergent and distinct fibrotic mechanisms

**DOI:** 10.1172/jci.insight.197579

**Published:** 2025-10-22

**Authors:** Yuanqing Yan, Taisuke Kaihou, Emilia Lecuona, Xin Wu, Masahiko Shigemura, Haiying Sun, Chitaru Kurihara, Ruli Gao, Felix L. Nunez-Santana, G.R. Scott Budinger, Ankit Bharat

**Affiliations:** 1 Department of Surgery, Division of Thoracic Surgery;; 2 Department of Biochemistry and Molecular Genetics; and; 3 Department of Medicine, Division of Pulmonary and Critical Care Medicine, Feinberg School of Medicine, Northwestern University, Chicago, Illinois, USA.

**Keywords:** Immunology, Pulmonology, Bioinformatics, Fibrosis, Transplantation

## Abstract

Chronic lung allograft dysfunction (CLAD) is the leading cause of mortality after lung transplantation, yet its molecular mechanisms remain poorly understood. To elucidate the pathogenesis of CLAD, we conducted a comprehensive single-cell transcriptomic analysis of CLAD lungs, integrating our generated datasets with approximately 1.6 million cells from 15 published studies of other fibrotic lung diseases. By applying pseudo-bulk approaches to mitigate batch effects, we identified molecular signatures specific to CLAD and those shared with idiopathic pulmonary fibrosis, COVID-19, and other fibrotic conditions. Our analysis revealed CLAD-specific cellular subsets including Fibro.AT2 cells, exhausted CD8^+^ T cells, and superactivated macrophages while suggesting that pathogenic keratin 17–positive, keratin 5–negative (KRT17^+^KRT5^−^) cells represent a common fibrotic mechanism across fibrotic lung diseases. Additionally, we performed donor-recipient cell deconvolution in lung allografts, uncovering distinct transcriptional programs and intercellular crosstalk between donor- and recipient-derived cells that drive allograft fibrosis. Recipient-derived stromal and immune cells showed enhanced pro-fibrotic and allograft rejection pathways compared with their donor counterparts. By leveraging insights from other fibrotic diseases to elucidate CLAD-specific mechanisms, our study provides a molecular framework for understanding CLAD pathogenesis and identifies potential therapeutic targets for this treatment-refractory condition.

## Abstract 2

An integrative single-cell transcriptomic analysis of fibrosis-associated lung diseases to uncover cellular and molecular signatures in chronic lung allograft dysfunction

## Introduction

Chronic lung allograft dysfunction (CLAD) represents the leading cause of mortality following lung transplantation, affecting up to 50% of recipients within 5 years and constituting the primary barrier to long-term allograft survival (1–3). Despite its clinical significance, CLAD remains one of the least understood fibrotic lung diseases, with no established disease-modifying therapies. CLAD manifests as progressive, irreversible decline in lung function exhibiting phenotypic overlap with other fibrotic lung diseases, including idiopathic pulmonary fibrosis (IPF) and postinfectious fibrosis (1–4). However, pathogenesis of CLAD occurs within the unique immunological context of transplantation, driven by alloimmune-mediated injury, chronic inflammation, and ischemia/reperfusion injury, resulting in complex cellular interactions between donor- and recipient-derived populations that distinguish it from other fibrotic conditions.

Elucidating pathogenesis of CLAD requires comprehensive molecular characterization; however, tissue availability and disease heterogeneity pose significant methodological challenges. While single-cell transcriptomic analyses have advanced our understanding of IPF and other fibrotic diseases, similar investigations in CLAD have been severely limited by tissue accessibility (5–12). Retransplantation for CLAD occurs in fewer than 3% of cases, restricting access to explanted CLAD lungs, while transbronchial biopsies suffer from sampling bias and may not capture the spatial heterogeneity of CLAD pathology. These constraints have resulted in a paucity of molecular studies examining CLAD pathogenesis and impeded the development of targeted therapeutics while preventing rational application of existing antifibrotic agents in patients with CLAD.

To address these limitations, we performed a comprehensive single-cell transcriptomic analysis centered on CLAD, integrating our generated CLAD datasets with approximately 1.6 million cells from 15 published studies encompassing IPF, non-IPF interstitial lung disease, chronic obstructive pulmonary disease (COPD), and COVID-19–associated fibrosis. This comparative approach enabled determination of CLAD-specific molecular signatures from conserved fibrotic pathways, while leveraging the unique transplant setting to perform donor-recipient cell deconvolution. Our primary objective was to elucidate pathogenesis of CLAD through identification of disease-specific cellular subsets, transcriptional programs, and intercellular communication networks that could serve as therapeutic targets while establishing an integrated reference atlas to inform understanding of fibrotic lung disease pathogenesis more broadly.

## Results

### Assembly of CLAD transcriptomic datasets with comparative fibrotic lung disease cohorts

To comprehensively characterize CLAD at the molecular level, we first assembled single-cell transcriptomic datasets from CLAD lungs and integrated these with comparative datasets from other fibrotic lung diseases. Given the scarcity of CLAD tissue, we generated single-cell RNA-Seq (scRNA-Seq) data from 4 CLAD explants obtained at retransplantation at Northwestern University (NU), including 3 cases of bronchiolitis obliterans syndrome (BOS) and 1 of restrictive allograft syndrome (RAS). All diagnoses were confirmed by pulmonary pathologist examination. We supplemented our CLAD cohort with previously published CLAD single-cell data from Khatri et al. (13), yielding 8 total CLAD samples for analysis.

To compare CLAD-specific molecular features with the broader fibrotic mechanisms, we developed SingleGEO, a computational toolkit for systematic identification of publicly available single-cell transcriptomic datasets (Supplemental Figure 1; supplemental material available online with this article; https://doi.org/10.1172/jci.insight.197579DS1). Using keywords related to pulmonary fibrosis, we identified 13 relevant studies encompassing IPF (5, 7, 14–18), non-IPF interstitial lung disease (ILD) (7, 16, 17, 19), COPD (5), ILD (7, 16, 17, 19), hypersensitivity pneumonitis (HP) (7, 17), nonspecific interstitial pneumonia (NSIP) (7), scleroderma (SCD) (15), and sarcoidosis (7) (Supplemental Figure 2A and Supporting Data Values). We additionally included COVID-19–associated fibrosis datasets (6, 18, 20–22), given the contemporary relevance and severe fibrotic phenotype, and generated single-nucleus RNA-Seq (snRNA-Seq) data from 3 COVID-19 explants obtained at transplantation from patients with fibroproliferative acute respiratory distress syndrome (6, 18, 20–22) (Supporting Data Values).

Our integrated analysis encompassed 1,576,567 cells total, including 141,734 newly generated cells from our CLAD and COVID-19 datasets. To validate spatial distribution of key cellular interactions identified in CLAD, we performed image-based spatial transcriptomics using the Xenium platform on formalin-fixed, paraffin-embedded lung samples from 3 patients with IPF, selected as a comparator given the shared fibrotic pathways between CLAD and IPF. This comprehensive dataset enabled systematic comparison of CLAD-specific versus conserved fibrotic mechanisms across multiple disease etiologies.

### Data integration and technical optimization for CLAD transcriptomic analysis

To enable robust molecular characterization of CLAD, we first addressed critical technical challenges in integrating diverse single-cell datasets. We performed data integration using scvi-tools (23), employing the Leiden clustering approach for cell identification (Supplemental Figure 2, A–D). Integration quality metrics confirmed successful batch effect removal (silhouette score: 0.72) while preserving biological variation (normalized mutual information: 0.79) (24), essential for distinguishing CLAD-specific signals from technical artifacts. We verified unbiased cell distribution across datasets and appropriate segregation into distinct populations (Supplemental Figure 2, E–G).

**Figure 1:**
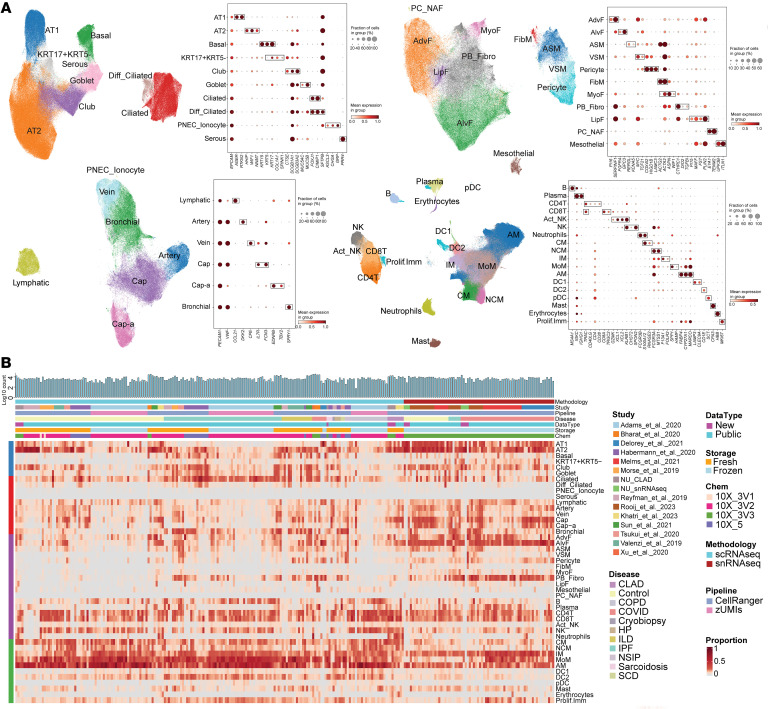
Integrated object of single-cell transcriptome from multiple datasets. (**A**) Uniform manifold approximation and projection (UMAP) plot displaying cell types, accompanied by a dot plot showing the expression levels of key marker genes. The expression level was *z*-score–transformed. AMs, alveolar macrophages; IM, interstitial macrophages; MoM, monocyte-derived macrophages. (**B**) Heatmap of cell type proportions across samples, where each row represents a cell type, and each column represents a sample. Diff_Ciliated, differentiating ciliated; PNEC_Ionocyte, pulmonary neuroendocrine cells and ionocyte; ASM, airway smooth muscle; VSM, vascular smooth muscle; FibM, fibromyocyte; LipF, lipofibroblast; PC_NAF, perichondrial or nerve-associated fibroblast; Cap, general capillary endothelial cells; Cap-a, capillary aerocyte; NCM, nonclassical monocytes; Act_NK, activated NK; pDC, plasmacytoid dendritic cells; Prolif.Imm, proliferative immune cells.

Cell type annotation was performed using established markers (5, 7, 25–28) and validated against the Human Lung Atlas using CellTypist (27, 29, 30), achieving high concordance (Figure 1A, Supplemental Figure 3, Supplemental Figure 4A, and Supporting Data Values). Cell abundance hierarchies aligned with expected lung composition across all datasets, with AMs consistently representing the predominant immune population (Figure 1B and Supporting Data Values). Notably, we observed method-dependent recovery biases, with scRNA-Seq detecting higher AM proportions than snRNA-Seq (Supplemental Figure 4B), a critical consideration given our CLAD samples were processed using scRNA-Seq.

Given the limited CLAD sample availability, maximizing data quality through rigorous technical assessment was paramount. We systematically evaluated multiple sources of technical variation that could confound CLAD-specific molecular signatures (Supplemental Figure 5). These included genome reference versions (outdated gene symbols like *SEPP1* versus *SELENOP*; Supplemental Figure 5A and Supplemental Figure 6, A and B), sequencing methods (scRNA-Seq versus snRNA-Seq, showing distinct transcriptomic profiles even in identical samples; Supplemental Figure 5B and Supplemental Figure 6, C and D), single-cell chemistry platforms (3′V2 versus 5′, identifying 274 chemistry-biased genes; Supplemental Figure 5C and Supplemental Figure 6, E and F), and data processing pipelines (revealing clear batch effects in pseudo-bulk analyses; Supplemental Figure 5D and Supplemental Figure 6G).

Understanding these technical factors was essential for accurate interpretation of CLAD-specific findings. For instance, the enrichment of cytoplasmic genes in scRNA-Seq versus nuclear genes in snRNA-Seq could artifactually influence pathway analyses. Similarly, chemistry-specific biases in genes like *SFTPC* (5′ chemistry) or *MALAT1* (3′V2 chemistry) required careful consideration when comparing CLAD samples with datasets using different platforms. By comprehensively characterizing these technical variables, we established a framework for distinguishing true CLAD-associated molecular features from technical artifacts, enabling reliable identification of disease-specific signatures despite the heterogeneous nature of the integrated datasets.

### Molecular signatures distinguishing CLAD from conserved fibrotic pathways

To identify molecular mechanisms specific to CLAD versus those shared across fibrotic lung diseases, we implemented rigorous batch correction to account for technical variation between datasets. Given the limited CLAD sample size, distinguishing true biological signals from technical artifacts was critical. We employed a pseudo-bulk approach with offsets, which effectively controls FDRs while maintaining statistical power comparable to generalized linear mixed models (31, 32), essential for analyzing our previous CLAD samples (Supplemental Figure 7A). We excluded datasets incompatible with CLAD analysis, such as Sun et al. (16), which used transplant rejection lungs as controls. After filtering, 37 cell types had sufficient representation for CLAD-comparative analysis.

We benchmarked 4 batch correction methods using AT2 cells, selecting ComBat-seq package for optimal performance with small sample sizes, which was crucial given our 8 CLAD samples (Supplemental Figure 7B). Validation against FACS-sorted AT2 cells from IPF verified high concordance in differential gene expression, supporting our methodology’s reliability for CLAD analysis (Supplemental Figure 8).

**Figure 2:**
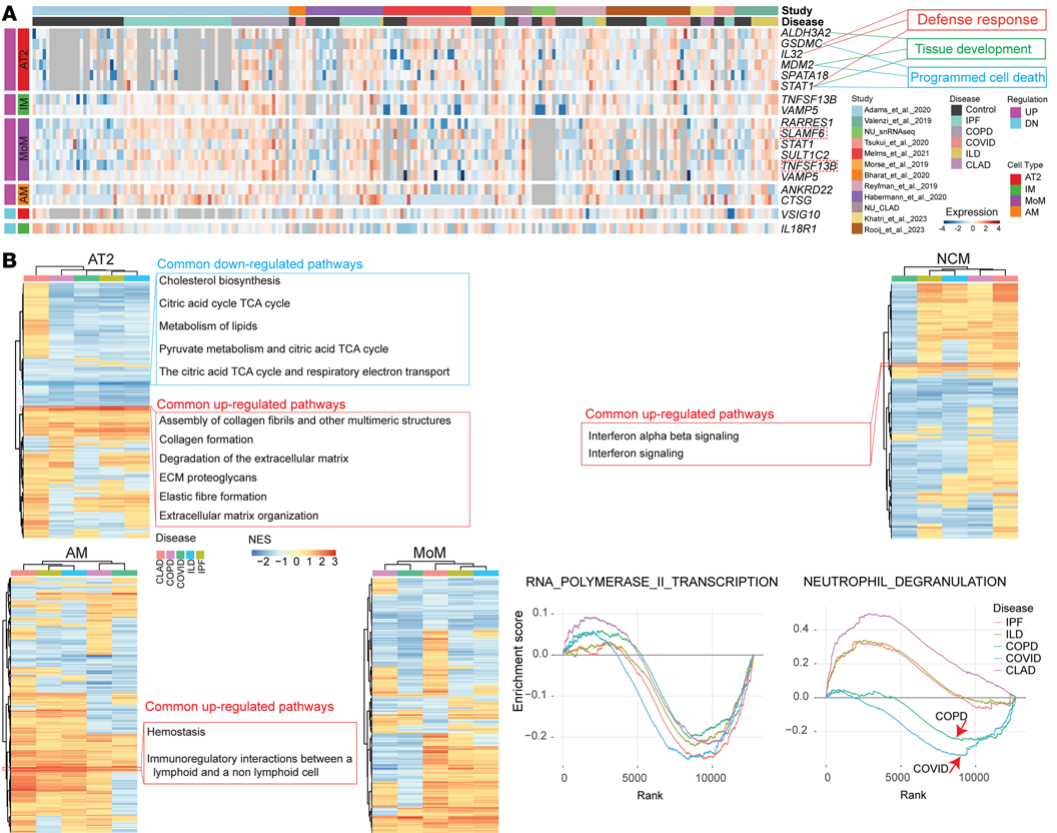
Shared molecular signature between CLAD and other disease etiologies. (**A**) Heatmap showing the expression levels of common genes across different cell types, with *z*-score–transformed values. Missing data are represented in gray. (**B**) Heatmap of commonly up- and downregulated Reactome pathways across the 5 disease etiologies. snRNA, single-nucleus RNA.

Applying this framework to CLAD samples revealed both unique and shared molecular features across 37 cell types. In CLAD AT2 cells, we identified 6 genes commonly upregulated across all fibrotic diseases, including defense response and programmed cell death pathways, suggesting these represent core fibrotic mechanisms rather than CLAD-specific alterations (Figure 2A and Supporting Data Values). CLAD also showed shared immune patterns: MoMs upregulated *SLAMF6* and *TNFSF13B*, genes critical for mononuclear cell differentiation and immune regulation; IMs expressed *VAMP5* and *TNFSF13B*; and AMs overproduced *ANKRD22* and *CTSG*.

Gene set enrichment analysis revealed both shared and distinct pathway activation patterns in CLAD compared with other fibrotic diseases (Figure 2B). In AT2 cells, CLAD shared upregulation of collagen formation and ECM proteoglycan pathways with other fibrotic conditions. Similarly, NCMs and AMs exhibited common enrichment of interferon signaling and immunoregulatory interaction pathways, respectively, highlighting conserved immune programs across fibrotic lung diseases. By contrast, CLAD displayed unique immune features: Neutrophil degranulation was substantially upregulated in CLAD (along with IPF and non-IPF ILD) but downregulated in COPD and COVID-19, suggesting distinct inflammatory mechanisms in allograft dysfunction. These results indicate that while CLAD shares both fibrotic and immune pathways with other conditions, it also harbors disease-specific immune alterations that could represent therapeutic targets unique to allograft dysfunction.

### CLAD-specific transcriptional signatures and comparative disease profiling

**Figure 3:**
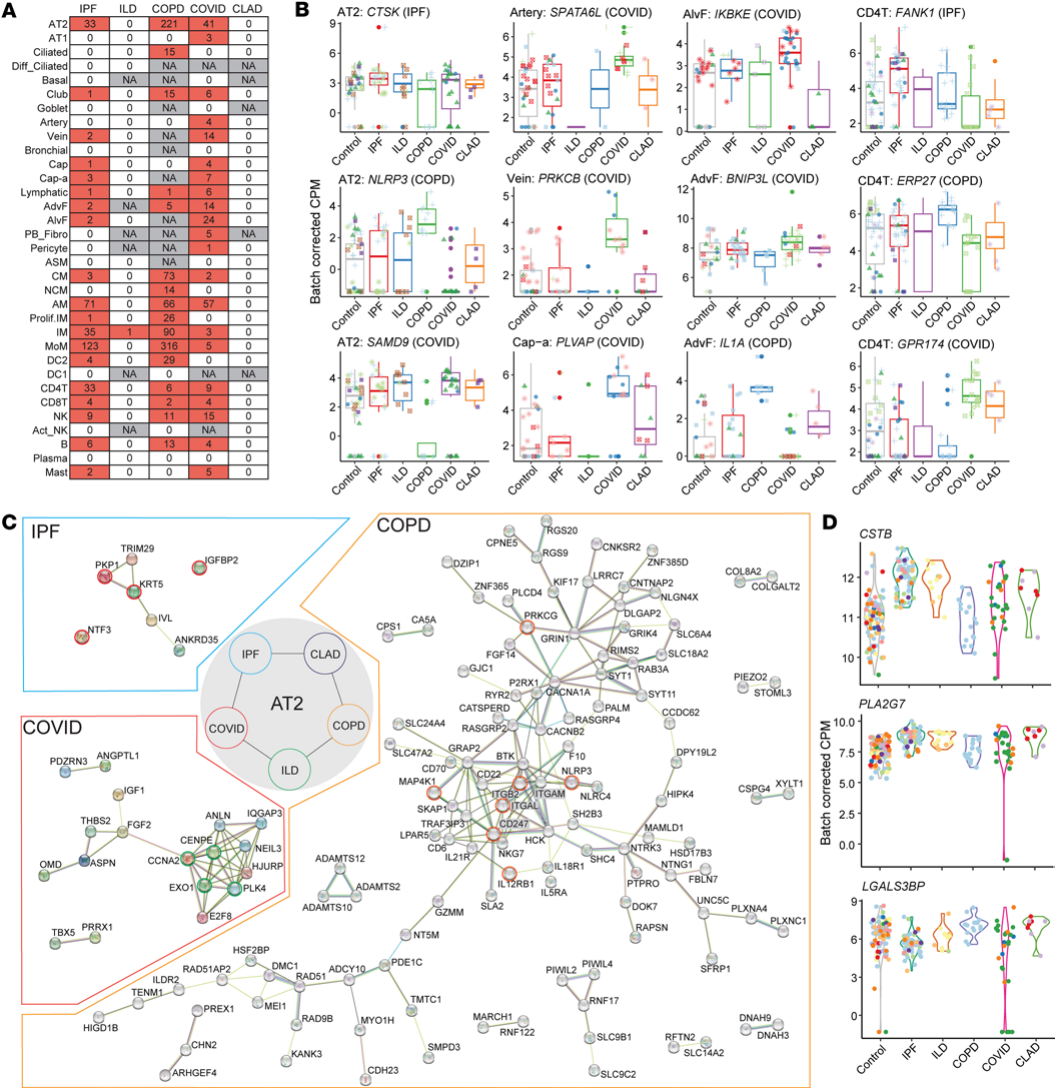
Transcriptomic divergence across disease etiologies. (**A**) Heatmap illustrating the number of differentially upregulated DUGs identified in each disease state, categorized by cell type. NA indicates that no analysis was performed due to insufficient sample size. (**B**) Box plots depicting the expression levels of selected DUGs across epithelial (AT2), endothelial, stromal (AlvF and AdvF), and immune (CD4T) compartments. Each dot represents an individual, with colors indicating the same dataset. Box plots show the interquartile range, median (line), and minimum and maximum (whiskers). CPM, counts per million. (**C**) Protein-protein interaction (PPI) network for DUGs in AT2 cells across IPF, COVID-19, and COPD. Genes involved in KRAS signaling in IPF are highlighted in red, genes in the G2M checkpoint pathway in COVID-19 in green, and genes in the allograft rejection pathway in COPD in orange. Genes without interactions or not part of signaling pathways are excluded. (**D**) Violin plot comparing expression levels of 3 representative genes across disease etiologies in pairwise comparisons. Violin plots show the full distribution of the data, with the width representing the density of values.

To identify molecular signatures unique to CLAD, we performed comprehensive transcriptional profiling using disease-unique gene (DUG) identification and pairwise comparisons across cell types. Despite rigorous analysis across 37 cell types, we did not detect significantly upregulated DUGs specific to CLAD (Figure 3A and Supporting Data Values). This may suggest that CLAD pathogenesis may involve dysregulation of shared fibrotic pathways rather than entirely unique molecular programs.

To contextualize CLAD within the broader fibrotic landscape, we characterized DUGs in comparator diseases. IPF showed *CTSK* overexpression in AT2 cells, COPD exhibited *NLRP3* activation, and COVID-19 displayed viral defense gene *SAMD9* upregulation (Figure 3B). These disease-specific signatures highlight distinct pathogenic mechanisms absent in CLAD, suggesting that allograft dysfunction may operate through different molecular pathways. Notably, protein-protein interaction (PPI) analysis revealed that COPD unexpectedly showed significant upregulation of allograft rejection gene sets (*q* value = 0.018), despite not involving transplantation, indicating potential shared immunological mechanisms with CLAD (Figure 3C and Supplemental Figure 9).

Given the absence of CLAD-specific DUGs, we employed pairwise comparisons to identify genes differentially expressed in CLAD relative to other diseases. Focusing on MoMs, critical mediators of fibrosis (33), we identified several CLAD-associated alterations (Supporting Data Values). *CSTB*, a cysteine protease inhibitor implicated in inflammation, was significantly upregulated in CLAD compared with controls and showed distinct expression patterns when compared with COPD (Figure 3D). Importantly, *PLA2G7*, encoding a phospholipase involved in inflammatory signaling, was significantly elevated in CLAD, with expression levels comparable to IPF but substantially higher than COPD. *LGALS3BP*, a galectin-binding protein involved in immune modulation, showed significantly higher expression in CLAD than IPF, suggesting enhanced immune activation in the allograft environment.

These pairwise comparisons reveal that while CLAD may not exhibit entirely unique gene programs, it displays distinctive expression patterns of immune and inflammatory mediators that differentiate it from other fibrotic diseases. The combination of elevated *CSTB*, *PLA2G7*, and *LGALS3BP* in CLAD MoM cells suggests a specific inflammatory-fibrotic signature in allograft dysfunction. These findings indicate that CLAD therapeutic strategies might benefit from targeting these differentially expressed inflammatory mediators, rather than searching for entirely novel pathways, and support the potential repurposing of existing antiinflammatory agents for CLAD treatment.

### Defining pathogenic signatures of KRT17+KRT5– cells in CLAD

To understand aberrant epithelial responses in CLAD, we characterized keratin 17–positive, keratin 5–negative (KRT17^+^KRT5^–^) cells, which represent pathogenic epithelial cells previously identified in fibrotic lungs (5–7) but not yet examined in the transplant context. These cells, known to localize to fibrotic niches (8, 10, 34), may represent a common pathogenic mechanism linking CLAD to other fibrotic diseases. Importantly, by incorporating CLAD samples into our analysis, we could determine whether these cells emerge in the unique immunological environment of lung allografts and identify their potential contribution to CLAD pathogenesis.

**Figure 4:**
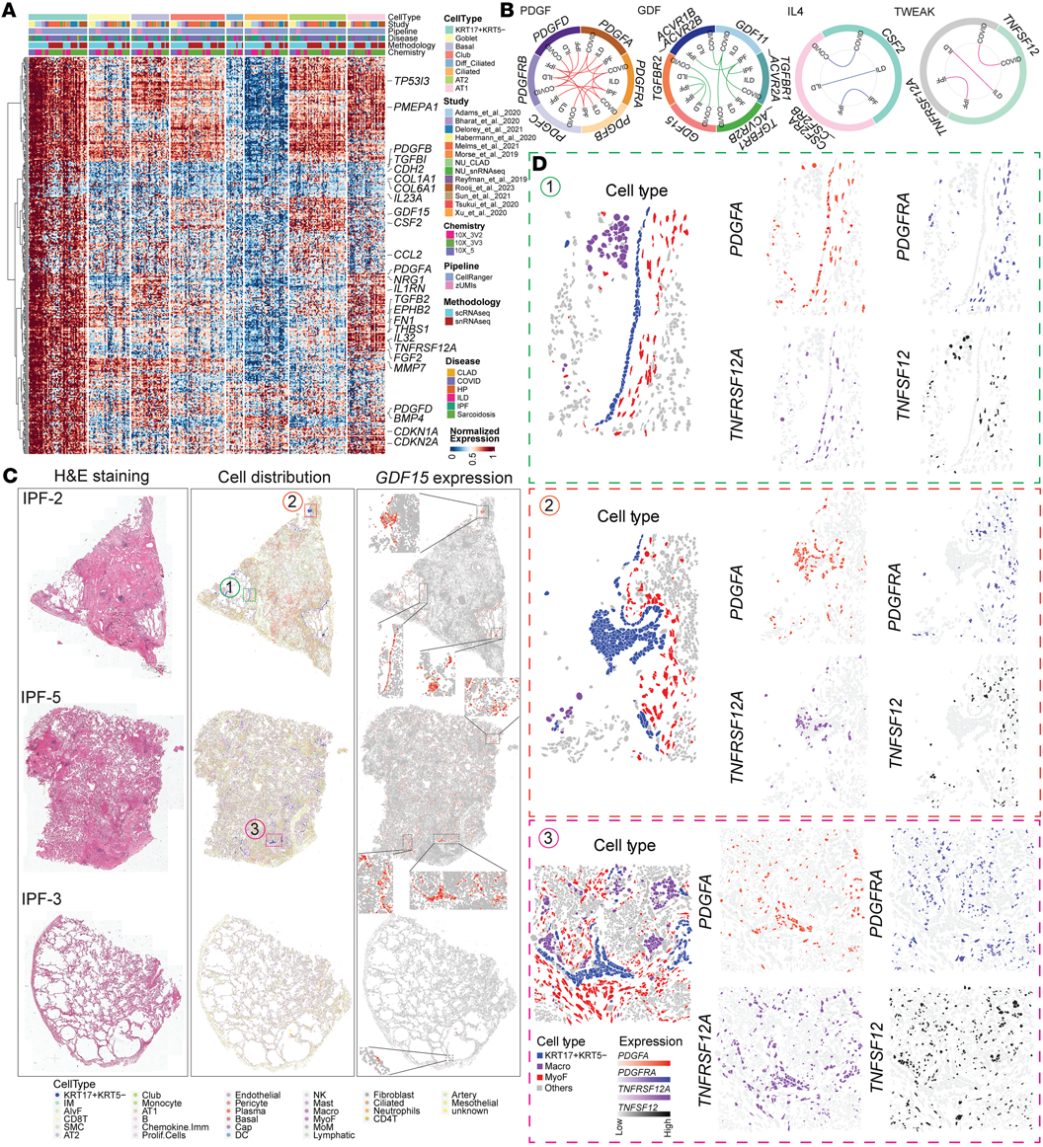
Core gene signature of KRT17^+^KRT5^–^ cells regardless of disease states and technical effects. (**A**) Heatmap of the expression levels of 360 core genes across different epithelial cell types, with selected gene symbols labeled. The average expression per gene was calculated for each sample from the normalized data matrix and further normalized per row, scaling the expression range between 0 and 1. Each column represents an individual sample. (**B**) Circle plot illustrating significant ligand-receptor pairs involved in PDGF, GDF, IL-4, and TWEAK signaling across diseases. Significant ligand-receptor interactions are connected by lines for each disease. (**C**) H&E staining and spatial distribution of cell types in 3 IPF lung samples. (**D**) Spatial niches enriched by KRT17^+^KRT5^–^cells, along with the expression of ligand-receptor pairs activated by these cells. The cellular composition varied across niches: niche1 contained 56 KRT17^+^KRT5^–^ cells, 59 MyoF cells, and 39 Macrophages; niche2 contained 218 KRT17^+^KRT5^–^ cells, 105 MyoF cells, and 12 Macrophages; and niche3 contained 172 KRT17^+^KRT5^–^ cells, 393 MyoF cells, and 195 Macrophages.

Leveraging our integrated dataset with CLAD samples, we defined a refined 360-gene core signature for KRT17^+^KRT5^–^ cells using pseudo-bulk RNA-Seq with rigorous batch correction (Figure 4A, Supporting Data Values, and Supplemental Figure 10A). The inclusion of CLAD data was critical for ensuring this signature captured features relevant to allograft dysfunction, not just native lung fibrosis. Top differentially expressed genes included *CDH2*, *CPA6*, *CAPN6*, and *SPINK1*, alongside established fibrosis mediators *COL1A1* and *PDGFA*. Epithelial-mesenchymal transition (EMT) emerged as the most enriched pathway (Supplemental Figure 10B), suggesting these cells undergo phenotypic transformation relevant to CLAD’s fibrotic remodeling. Pathway analysis revealed enrichment for collagen formation, ECM organization, and multiple cell-cell communication networks, processes central to CLAD pathogenesis.

Analysis of KRT17^+^KRT5^–^ cell interactions revealed signaling mechanisms potentially targetable in CLAD. While our CLAD cohort size limited direct cell-cell interaction analysis, the conserved signaling pathways identified across IPF, non-IPF ILD, and COVID-19 provide insights into likely CLAD mechanisms (Supplemental Figure 10, C–E). KRT17^+^KRT5^–^ cells consistently activated pro-fibrotic pathways (PDGF, TGF-β, GDF) and immune-regulatory signals (IL-4), both critical in CLAD progression. The PDGF pathway, with KRT17^+^KRT5^–^ cells overexpressing *PDGFA*, *PDGFB*, and *PDGFD*, represents a particularly attractive target given existing FDA-approved PDGFR inhibitors like nintedanib (Figure 4B). Similarly, GDF15 overexpression and CSF2-mediated signaling suggest additional therapeutic avenues for CLAD.

Spatial transcriptomic validation in IPF lungs, selected as a CLAD surrogate given shared fibrotic mechanisms, verified KRT17^+^KRT5^–^ cells cluster within fibrotic foci with enriched myofibroblast and immune cell populations (Figure 4, C and D, and Supplemental Figure 11). The localized PDGFA/PDGFRA signaling to adjacent myofibroblasts and TNFRSF12A-mediated immune crosstalk demonstrate these cells orchestrate local fibrotic niches through paracrine signaling. These spatial relationships likely occur similarly in CLAD lungs, where KRT17^+^KRT5^–^ cells could contribute to both airway-centered fibrosis (BOS phenotype) and parenchymal scarring (RAS phenotype).

The identification of KRT17^+^KRT5^–^ cells sharing core signatures across CLAD and other fibrotic diseases has important therapeutic implications. Rather than requiring entirely novel treatments, patients with CLAD might benefit from existing antifibrotic strategies targeting these cells, such as PDGFR inhibition or anti-EMT approaches. Our findings suggest KRT17^+^KRT5^–^ cells represent a convergent pathogenic mechanism in lung fibrosis, making them attractive therapeutic targets for CLAD, where treatment options remain severely limited.

### Molecular dissection of CLAD pathogenesis at single-cell resolution

#### Donor-recipient chimerism reveals potentially unique CLAD cellular architecture.

**Figure 5:**
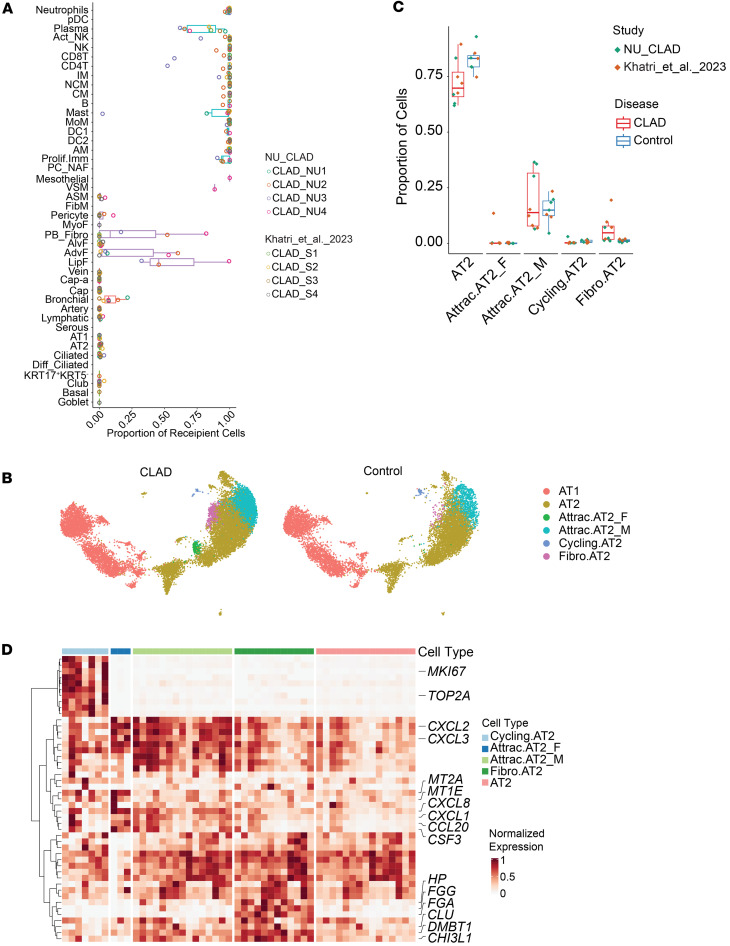
Single-cell transcriptomic landscape of epithelial cells in CLAD. (**A**) Box plot of the proportion of recipient cells for each cell type. (**B**) UMAP visualization of different subsets of alveolar epithelial cells between CLAD and control groups. (**C**) Box plot of the relative abundance of each AT2 subset within individual samples. Box plots show the interquartile range, median (line), and minimum and maximum (whiskers). (**D**) Heatmap illustrating the normalized gene expression of top 10 marker genes for each AT2 subset. Normalization was performed within each individual to a 0–1 range. Marker genes were selected based on differential expression analysis of scRNA-Seq data using FindAllMarkers in Seurat with an adjusted *P* value cutoff of 0.05.

To elucidate CLAD pathogenesis, we performed comprehensive single-cell analysis of end-stage CLAD lungs obtained at retransplantation, representing a rare opportunity given that fewer than 3% of patients with CLAD undergo retransplantation. By leveraging genetic variant calling to distinguish donor from recipient cells, we uncovered the chimeric cellular landscape unique to transplanted lungs (Figure 5A and Supporting Data Values).

The pattern of chimerism revealed fundamental insights into CLAD pathobiology. While epithelial and endothelial compartments remained predominantly donor derived, preserving allograft structure, the stromal compartment (AdvF, LipF, PB-Fibro) exhibited extensive recipient infiltration. Most strikingly, immune cells were overwhelmingly recipient derived, except for residual donor plasma cells. This architecture with donor structural cells surrounded by recipient immune and stromal cells creates a unique pathogenic microenvironment absent in native lung fibrosis.

#### CLAD-specific epithelial reprogramming links coagulation to fibrosis.

Analysis of the epithelial compartment identified 3 AT2 subsets emerging specifically in CLAD (Figure 5, B–D, and Supplemental Figure 12). Most notably, Fibro.AT2 cells, characterized by massive upregulation of coagulation cascade genes (*FGG*, *FGA*, *HP*), appeared consistently across all CLAD samples. While present in other fibrotic diseases, their universal presence in CLAD suggests coagulation dysregulation as a core pathogenic mechanism in allograft dysfunction.

Two sex-specific subsets, Attrac.AT2_M (male, *DDX3Y*^+^) and Attrac.AT2_F (female, *XIST*^+^), exhibited distinct chemokine profiles driving immune recruitment, with maximal enrichment for allograft rejection pathways (Supplemental Figure 12, B–E). This sex-specific epithelial response may explain reported sex disparities in transplant outcomes and suggests personalized therapeutic approaches based on donor-recipient sex matching.

#### Recipient cells drive inflammatory amplification across compartments.

**Figure 6:**
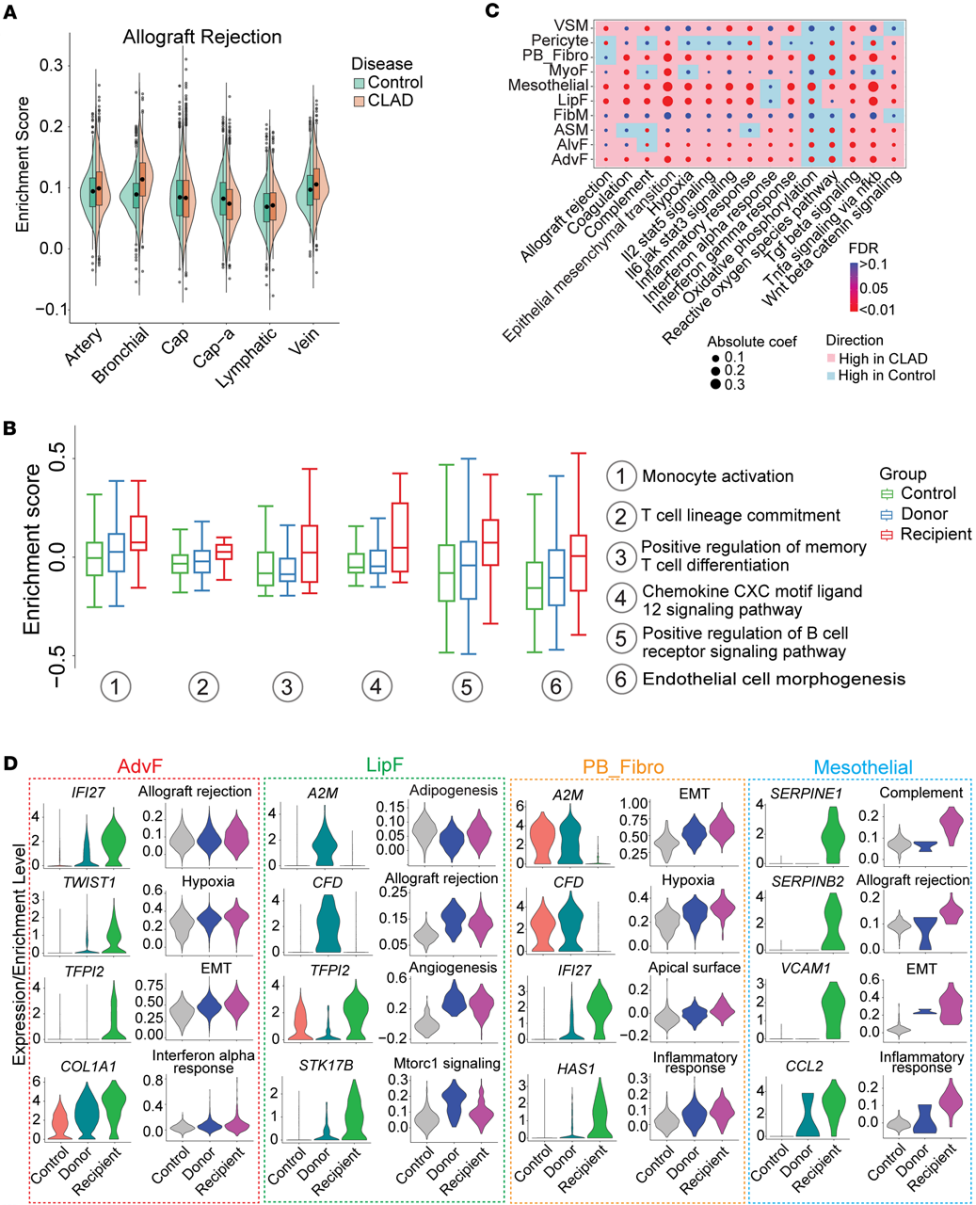
Single-cell transcriptomic landscape of endothelial and stromal cells in CLAD. (**A**) Violin plot of allograft rejection scores among different endothelial cell types. Violin plots show the full distribution of the data, with the width representing the density of values. Adjusted *P* value was obtained from the Wilcoxon rank-sum test for the comparison between CLAD and control. (**B**) Box plot of biological process scores in bronchial endothelial cells of scRNA-Seq data, distinguishing donor and recipient cells. Box plots show the interquartile range, median (line), and minimum and maximum (whiskers). A linear mixed effects model was fitted using the lmerTest package in R, with different datasets treated as a random effect. Pairwise comparisons were conducted using Tukey’s post hoc analysis. Biological processes with an adjusted *P* < 0.05 were considered statistically significant. (**C**) Dot plot of enrichment scores for different pathways across various stromal cells. The pink background indicates higher scores in the CLAD group, while the light blue background indicates higher scores in the control group. Dot size is proportional to the absolute coefficient value from the linear regression, and filled dot color corresponds to different FDR values. (**D**) Violin plot of significant genes and pathways between donor and recipient cells across different stromal cell types. The selected genes were the significant ones with FDR < 0.05 calculated from differential expression analysis of scRNA-Seq data using FindAllMarkers in Seurat. For the pathway comparison between 3 groups, the Kruskal-Wallis analysis followed by pairwise Wilcoxon rank-sum tests at cell level was performed. The Benjamini-Hochberg (BH) method was used for *P* value adjustment, and pathways with FDR < 0.05 were selected for plotting.

Comparative analysis of donor versus recipient cells within identical cell types revealed fundamental differences in inflammatory activation. Recipient-derived bronchial endothelial cells — unexpectedly present in CLAD samples — showed dramatic upregulation of monocyte activation, T cell differentiation, and chemokine signaling compared with donor endothelium (Figure 6, A and B, and Supplemental Figure 13). This challenges the paradigm that endothelial rejection targets only donor cells.

Within stromal compartments, recipient-derived fibroblasts exhibited a hyperactivated phenotype with enhanced expression of *IFI27* (immune response), *TWIST1* (EMT), *TFPI2* (coagulation), and *COL1A1* (fibrosis) compared with donor fibroblasts (Figure 6, C and D). These recipient stromal cells showed the strongest allograft rejection signatures, positioning them as active mediators rather than bystanders in CLAD progression.

#### Exhausted T cells define CLAD-specific adaptive immunity.

**Figure 7:**
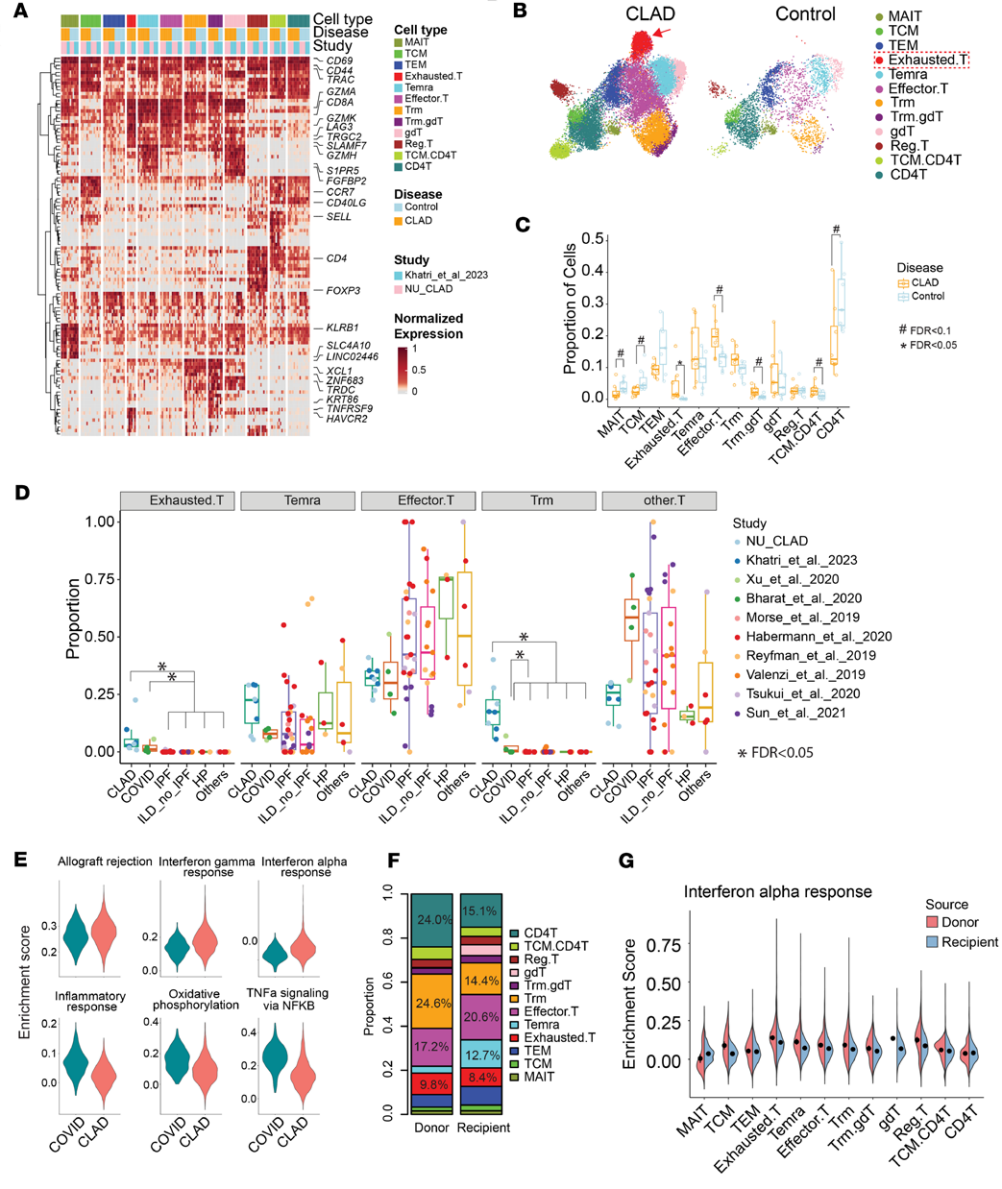
Single-cell transcriptomic landscape of lymphoid cells in CLAD. (**A**) Heatmap of gene expression across T cell subtypes. The average expression per gene was calculated for each individual and further normalized within each row to a 0–1 range. (**B**) UMAP visualization of T cell subtypes, separated by CLAD and control groups. (**C**) Box plot showing the proportion of different T cell subtypes. Box plots show the interquartile range, median (line), and minimum and maximum (whiskers). (**D**) Box plot showing the proportion of each CD8^+^ T cell subtype per sample across multiple diseases. (**E**) Violin plot of pathway enrichment scores comparing CLAD and COVID-19 in exhausted CD8^+^ T cells. Violin plots show the full distribution of the data, with the width representing the density of values. (**F**) Stacked bar chart illustrating the distribution of T cell subtypes within donor and recipient cell groups. (**G**) Violin plot comparing IFN-α response scores across T cell subtypes in donor and recipient cells: Trm (tissue-resident memory T cells), TEM (effector memory T cells), TCM (central memory T cells), γδT (gamma delta T cells), Reg.T (regulatory T cells), Effector.T (effector CD8^+^ T cells), MAIT (mucosal-associated invariant T cells), Temra (terminally differentiated effector memory T cells). For comparisons between 2 groups, the Wilcoxon test was used. For comparisons involving more than 2 groups, the Kruskal-Wallis test was followed by pairwise Wilcoxon rank-sum tests. *P* values were adjusted using the BH method, and FDR < 0.05 was considered statistically significant.

Among lymphoid populations, exhausted CD8^+^ T cells emerged as a defining feature of CLAD, consistently enriched across all samples (Figure 7, A–C, and Supplemental Figure 14A). These cells uniquely coexpressed exhaustion markers (*HAVCR2*, *LAG3*, *PD1*) alongside active cytotoxic programs (*TNFRSF9*, *GZMK*, *GZMH*), validated by immunofluorescence (Supplemental Figure 14, B–D, and Supplemental Figure 15).

Exhausted T cells appeared exclusively in CLAD and COVID-19 among all fibrotic diseases analyzed but with distinct functional profiles (Figure 7D). CLAD Exhausted.T cells showed maximal allograft rejection and interferon responses, whereas COVID-19 cells exhibited oxidative phosphorylation and TNF-α activation (Figure 7E). This CLAD-specific exhaustion signature likely reflects chronic alloantigen exposure unique to transplantation.

Remarkably, donor-derived T cells — passenger lymphocytes persisting in allografts — maintained significantly higher interferon responses than recipient T cells (Figure 7, F and G), suggesting sustained activation despite being outnumbered, potentially perpetuating allograft injury.

#### Superactivated macrophages orchestrate CLAD inflammation.

**Figure 8:**
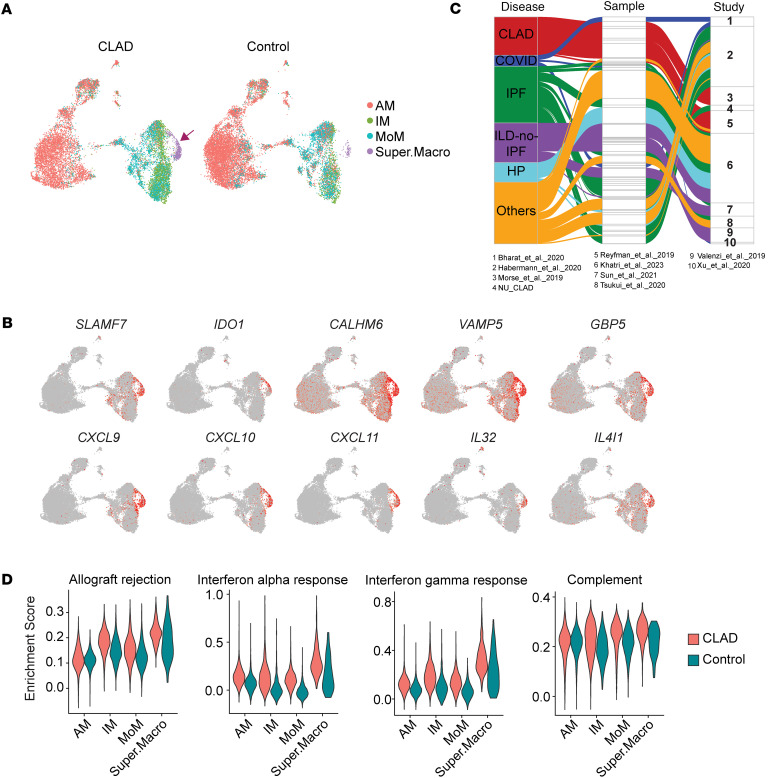
Single-cell transcriptomic landscape of myeloid cells in CLAD. (**A**) UMAP visualization of macrophage subsets, separated by CLAD and control groups. Arrow shows where the cell type is. (**B**) Feature plot highlighting the overexpression of key genes in the Super.Macro subset. (**C**) Alluvial plot illustrating the proportion of Super.Macro within the IM and MoM populations per sample across different fibrotic disease types. Only scRNA-Seq datasets were included in this analysis, excluding Adam et al. 2020 due to differences in data processing pipelines. (**D**) Violin plot of pathway enrichment scores across different macrophage subsets.

Within myeloid compartments, we identified superactivated macrophages (Super.Macro) as a distinct population enriched in CLAD, expressing *SLAMF7*, a marker of extreme activation (35) (Figure 8, A and B). These cells exhibited unprecedented upregulation of immunoregulatory genes (*IDO1*, *CALHM6*, *VAMP5*) and chemokines (*CXCL9*, *CXCL10*, *CXCL11*), creating the pro-inflammatory milieu characteristic of CLAD (Figure 8B).

While Super.Macro cells appeared in other fibrotic diseases, their abundance and activation profile peaked in CLAD with maximal allograft rejection pathway enrichment (Figure 8, C and D). Classical monocytes in CLAD showed complement activation and adhesion programs, while NCMs exhibited apoptotic signatures, revealing compartment-specific myeloid dysfunction (Supplemental Figure 16).

#### Donor-recipient crosstalk creates pathogenic signaling circuits.

**Figure 9:**
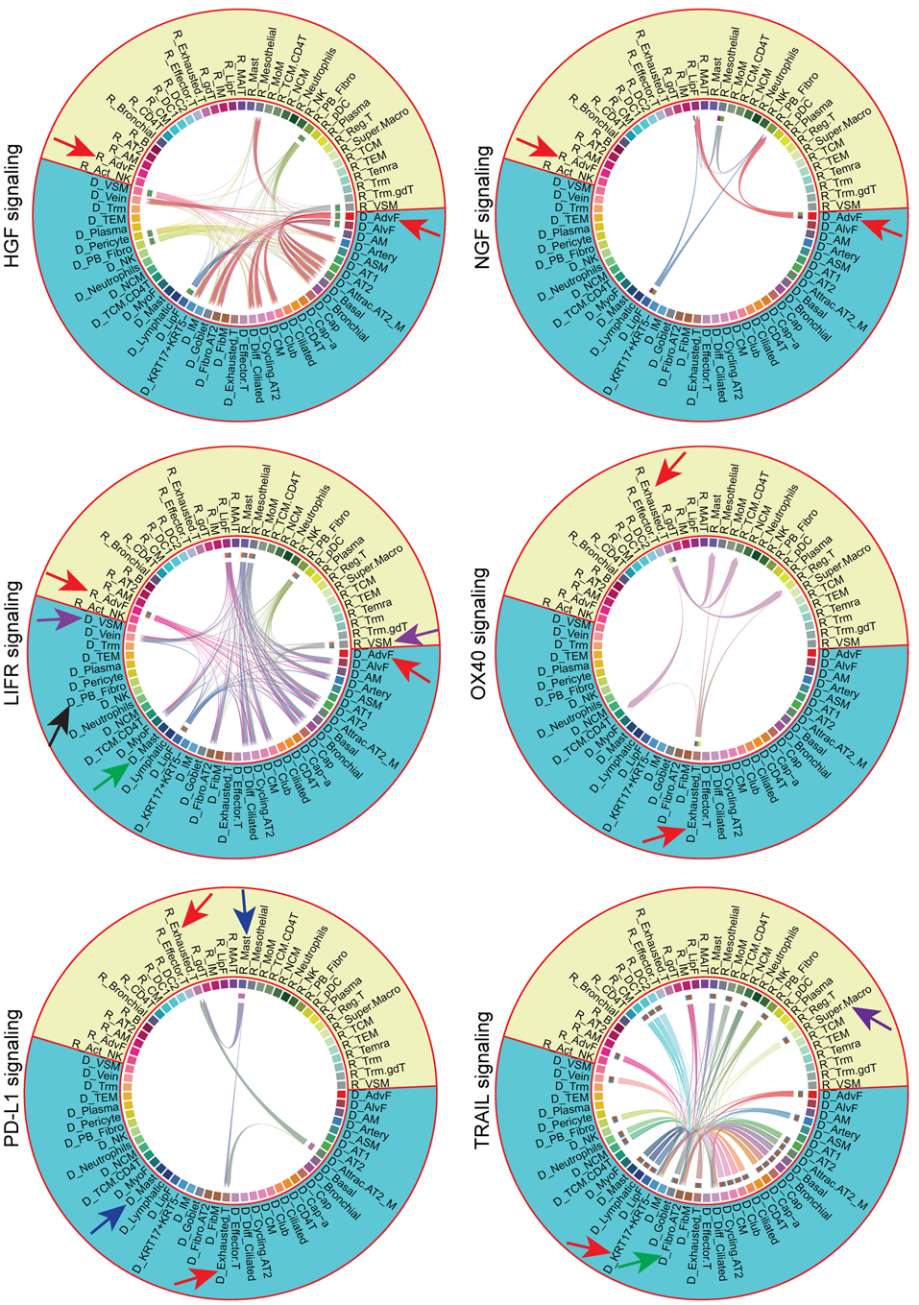
Donor-recipient crosstalk in CLAD. Circle plot depicting the signal flow of cell-cell interactions in CLAD, distinguishing donor from recipient cells.

Cell interaction analysis revealed an unexpected signaling hierarchy unique to CLAD’s chimeric environment (Figure 9). Donor adventitial fibroblasts emerged as major regulators, sending signals to CLAD-specific populations (Fibro.AT2, KRT17^+^KRT5^–^ cells) via the HGF-MET pathway, positioning donor stroma as active participants in allograft dysfunction rather than passive victims.

Critical pathogenic circuits included (a) Exhausted.T cells regulating CD4^+^ T cells through OX40, perpetuating T cell dysfunction; (b) Super.Macro cells targeting epithelium via TRAIL, linking inflammation to epithelial injury; and (c) failed immune checkpoints through programmed cell death ligand 1/programmed cell death 1 (PD-L1/PD-1) interactions between Attrac.AT2 and Exhausted.T cells (Supplemental Figure 17). These donor-recipient signaling networks, impossible in native lung diseases, define CLAD as a disease of cellular miscommunication with specific therapeutic targets (HGF-MET, TRAIL, PD-L1/PD-1) for interrupting progression.

## Discussion

This study represents a comprehensive molecular characterization of CLAD, leveraging single-cell transcriptomic analysis of 8 CLAD lungs integrated with approximately 1.6 million cells from other fibrotic diseases. By overcoming significant technical challenges, including tissue scarcity and batch effects, we identified both CLAD-specific molecular features and conserved fibrotic mechanisms, providing critical insights into allograft dysfunction pathogenesis. Our ability to distinguish donor from recipient cells within CLAD lungs revealed potentially previously unrecognized cellular subsets (Fibro.AT2, Exhausted.T, Super.Macro) and pathogenic cell-cell interactions specific to the transplant environment. These findings establish a molecular framework for understanding CLAD, or other fibrotic lung diseases, and identify actionable therapeutic targets for this treatment-refractory condition.

The identification of CLAD-specific cellular subsets provides insights into lung allograft dysfunction. Fibro.AT2 cells, characterized by massive coagulation cascade upregulation (*FGG*, *FGA*, *HP*), appeared universally in CLAD samples, suggesting coagulation dysregulation as an important pathogenic mechanism. This finding aligns with clinical observations of increased thrombotic events in CLAD and suggests anticoagulation strategies merit investigation. The sex-specific Attrac.AT2 subsets (Attrac.AT2_M and Attrac.AT2_F) provide molecular evidence for reported sex disparities in transplant outcomes (36), potentially informing personalized therapeutic approaches based on donor-recipient sex matching. Exhausted.T cells found in CLAD likely reflect chronic alloantigen exposure unique to transplantation. Their coexpression of exhaustion markers with active cytotoxic programs suggests failed immune regulation, a therapeutic opportunity through checkpoint modulation. Superactivated macrophages, while present in other fibrotic diseases, showed maximal activation in CLAD with unprecedented chemokine production (*CXCL9*, *CXCL10*, *CXCL11*), creating the inflammatory milieu characteristic of allograft dysfunction.

Donor-recipient cell deconvolution revealed fundamental insights impossible to obtain from native lung diseases. The discovery that recipient-derived cells infiltrate not only immune but also stromal and endothelial compartments challenges current paradigms of allograft rejection. Recipient bronchial endothelial cells, present in CLAD samples, showed dramatic inflammatory activation compared with donor endothelium, suggesting endothelial chimerism actively contributes to, rather than protects against, rejection (37–40). Recipient stromal cells exhibited hyperactivated phenotypes with maximal allograft rejection signatures, positioning them as active mediators of CLAD progression. This cellular chimerism creates unique pathogenic opportunities in which donor structural cells and recipient immune/stromal cells engage in aberrant dialogs impossible in native lungs, defining CLAD as fundamentally distinct from other fibrotic diseases despite phenotypic similarities (1–3, 41).

The absence of CLAD-specific upregulated disease-unique genes, despite rigorous analysis, provides important biological insights. Rather than operating through entirely novel pathways, CLAD appears to dysregulate shared fibrotic mechanisms in unique combinations within the alloimmune environment. This finding may suggest that patients with CLAD might benefit from existing antifibrotic agents combined with immunomodulation rather than requiring entirely novel drugs. Our pairwise comparisons revealed CLAD-specific expression patterns of inflammatory mediators (*CSTB*, *PLA2G7*, *LGALS3BP* in MoMs), suggesting targeted antiinflammatory approaches. The unexpected finding that COPD shows allograft rejection pathway activation despite not involving transplantation hints at shared immunological mechanisms that could inform CLAD treatment strategies.

By incorporating CLAD data into KRT17^+^KRT5^–^ cell analysis, we established these pathogenic epithelial cells as a convergent mechanism linking CLAD to other fibrotic diseases. The 360-gene signature we defined, including PDGF, GDF, and TGF-β pathway components, suggests patients with CLAD could benefit from existing therapies targeting these cells, such as nintedanib or pirfenidone (42–48). The spatial validation showing KRT17^+^KRT5^–^ cells orchestrating fibrotic niches through PDGFA/PDGFRA signaling provides rationale for PDGFR inhibition in CLAD. While our CLAD sample size limited direct cell-cell interaction analysis, the conserved signaling patterns across other fibrotic diseases suggest these mechanisms likely operate similarly in CLAD lungs.

Our technical efforts were essential for extracting meaningful CLAD insights from limited samples. The systematic evaluation of batch effects including genome versions, sequencing methods, chemistry platforms, and processing pipelines enabled distinction of true CLAD biology from technical artifacts. This methodological rigor is particularly critical for rare diseases like CLAD, where sample acquisition is challenging. The pseudo-bulk approach with ComBat-seq correction preserved statistical power while controlling false discoveries, essential given our 8 CLAD samples. These technical advances provide a framework for future CLAD studies and rare disease research more broadly.

This study has limitations that highlight challenges in CLAD research. The small sample size (8 CLAD lungs) reflects the rarity of retransplantation but limits statistical power for detecting CLAD-specific genes. The heterogeneity within CLAD (3 BOS, 1 RAS) may mask subtype-specific signatures that larger cohorts could reveal. Lack of clinical metadata in published datasets prevented correlation of molecular findings with outcomes. Batch correction, while necessary, may obscure subtle biological signals. Our findings require functional validation, particularly of the potentially novel cell subsets and signaling circuits. Future work should include expanded CLAD cohorts, longitudinal sampling to capture disease progression, and experimental validation of therapeutic targets in model systems.

In conclusion, this comprehensive molecular characterization of CLAD reveals it as a unique disease entity characterized by specific cellular subsets, donor-recipient chimerism, and aberrant cell-cell communication within the alloimmune environment. While sharing core fibrotic machinery with other lung diseases, CLAD exhibits distinct cellular and molecular features that provide immediate therapeutic opportunities. The identification of targetable pathways (HGF-MET, PDGF, GDF) and disease-specific cell populations offers hope for developing effective treatments for this devastating complication of lung transplantation. Our integrated dataset and analytical framework provide a foundation for future CLAD research and demonstrate how comparative transcriptomics can illuminate rare disease pathogenesis when direct studies are limited by tissue availability.

## Methods

### Sex as a biological variable.

Both male and female human samples were included in this study. Data were analyzed without stratification by sex, as the study was not designed to evaluate sex-specific differences. However, inclusion of both sexes increases the generalizability of the findings.

### Developing a computational toolkit of SingleGEO for single-cell database querying.

To facilitate the database query and ensure consistent analysis procedure, we developed a computational toolkit, SingleGEO. SingleGEO consists of 3 main modules: metadata query, sequencing data download, and downstream integrative analysis. For metadata query, SingleGEO is equipped with a built-in function to filter the potential single-cell studies. The Structural Query Language search engine is used to search and filter the study. Multiple keywords can be provided to aid the query, and the program searches the contents of title, summary, and overall design of each National Center for Biotechnology Information Gene Expression Omnibus (NCBI GEO) Series record (GSE). The returned query result consists of the key information of each GSE/Sample record. By establishing the bridge with NCBI GEO from R computing environment, single-cell sequencing data deposited in the GSE supplementary files are downloaded and analyzed (49).

### Identification and preprocessing of human lung disease–focused transcriptomic datasets.

We utilized SingleGEO and manual examination to search for and download publicly available single-cell transcriptome datasets in multiple lung diseases. We focused on datasets with the availability of all cell types from epithelial, endothelial, stromal, and immune compartments. Datasets targeting specific cell types or compartments were excluded to reduce computational demands. Similarly, datasets focusing on blood, bronchoalveolar lavage, or nonlung tissues were omitted. We also excluded datasets derived from isolated human cells cultured in vitro. For each selected dataset, we constructed a Seurat object and performed quality control assessments. When metadata were provided by the dataset submitters, cells with annotated cell types were considered high-quality and used for further analysis. In the absence of metadata, we filtered out low-quality cells or doublets based the number of features being less than 200 or greater than 7,500, the number of unique molecular identifiers being less than 400 or greater than 40,000, and the percentage of mitochondrial genes being larger than the designated threshold (10% for the majority of datasets; exceptions were made when a different cutoff was used in the original manuscript). Raw count data that passed quality control, along with the corresponding metadata, were exported for further data integration analysis.

### Single-cell transcriptome data integration by scvi-tools.

We integrated all targeted single-cell transcriptome data, along with 1 snRNA-Seq dataset of COVID-19 samples and 1 scRNA-Seq dataset of CLAD generated in our institute, using scvi-tools (23). Scvi-tools is a Python library designed for deep probabilistic analysis of single-cell omics data. It trains models efficiently through graphic processing unit computing with minibatching and implements features that simultaneously remove unwanted variation due to multiple nuisance factors (23). We set each individual sample as a batch for batch effect correction. Most datasets were processed using the Cell Ranger pipeline. However, the dataset from the Adams et al. study was analyzed with the zUMIs pipeline (v2.0), which provided gene features as a mix of HGNC and Ensembl_GeneID. To maintain consistency with other datasets, we removed genes labeled as ensemble ID without HGNC in the Adams et al. dataset for further analysis. We selected the top 3,000 highly variable genes to facilitate downstream dimension reduction and data integration procedures. Last, we used UMAP visualization of the scvi latent space to evaluate the low-dimensional embeddings of cells. The quality of data integration was examined by assessing cell distribution bias across datasets within clusters, variations in gene and unique molecular identifier (UMI) counts among clusters, and the separation of cells into distinct populations. Additionally, we used the scIB package to compute key evaluation metrics (24). For batch correction, we applied the silhouette score, which measures how well cells from the same batch cluster together compared with cells from different batches. For biological conservation, we used normalized mutual information to assess the consistency between clustering results and annotated cell labels.

### Cell type annotation.

We classified cells into epithelial, endothelial, immune, and stromal compartments based on gene expression levels of *EPCAM*, *PECAM1*, and *PTPRC*, extracting cells from each compartment for fine-resolution subclustering analysis. To optimize biological interpretability, we iteratively adjusted clustering resolution to best capture cell type distinctions, ensuring alignment with known populations. Differential gene expression analysis was then conducted between cell clusters, with annotations guided by established markers from previous studies (Supporting Data Values) (5, 7, 25–28). During annotation, clusters containing doublets, low-quality cells, or contaminating cardiac muscle cells were excluded from further analysis. Additionally, quality control measures — including assessments of gene count, UMI count, and mitochondrial gene percentage — were applied to filter out low-quality clusters before downstream analysis. Where possible, we compared our annotations with those from the original published datasets we integrated, ensuring consistency and robustness. To further validate our classifications, we analyzed average gene expression per sample from the normalized data matrix to assess robustness across conditions. Finally, we leveraged well-annotated cell types from the Human Lung Atlas (https://data.humancellatlas.org/) as a reference, predicting cell types in our integrated dataset to evaluate concordance with the Human Lung Atlas annotations.

### Testing the impact of reference genome build on transcriptomic profile.

We inferred the reference genome patch release version of each study based on the detection of *SELENOP*, a gene that was previously labeled as *SEPP1* in the release version prior to September 21, 2016. We defined the reference genome as the outdated one if the gene symbol of *SEPP1* was used. In addition, we examined the presence of genes in each study and converted such information to a binary data for the analysis. Jaccard distance was calculated, and we used hierarchical clustering with complete linkage to evaluate the similarity between studies.

### Evaluating the impact of sequencing profiling methods on gene signature differences.

To assess the impact of sequencing profiling methods on gene signature differences, we conducted the following analyses: examining the impact on AT2 using 7 control samples from 3 NU studies (Bharat et al., ref. 6; NU_snRNAseq and NU_CLAD), evaluating differentially expressed genes in CD8^+^ T cells from 1 COVID-19 sample (PMB1) sequenced by both scRNA-Seq and snRNA-Seq methods from our institute, and investigating the 3 COVID-19 lungs with simultaneous scRNA-Seq and snRNA-Seq from Delorey et al. dataset (22). For the subclustering of cell types, we extracted the expression count matrix and used Harmony approach for the data integration (50).

### Identification of influential genes impacted by different single-cell chemistries.

To study the impact of single-cell chemistries on gene expression profile, we used the control samples from the datasets of Habermann et al. (10X 5′) (7) and Reyfman et al. (10X 3V′2) (17) for the analysis. Both datasets were profiled by scRNA-Seq, used outdated reference genome, and were processed with Cell Ranger pipeline (10x Genomics). We selected AT2 in this study and calculated the average expression level for each gene. A linear regression was used to fit the averaged value for each gene between these 2 single-cell chemistries. We then calculated the Cook’s distance (Cook’s D), and the genes with Cook’s D larger than 3 times of the mean Cook’s distance were regarded as the influential genes.

### Pseudo-bulk gene count and the impact of pipelines on gene signature differences.

From the integrated object, we computed the pseudo-bulk gene count for each cell type within each sample by summing all the raw counts of each cell. For further analysis, we only retained samples with a cell number greater than 25. To examine the impact of processing pipelines on gene signature differences, we restricted our analysis in AMs (the most abundant cells in immune compartment), which were sequenced by scRNA-Seq method. We then removed the ribosomal protein genes, filtered out mitochondrial genes, and kept only protein-coding genes for the further analysis. We normalized the gene expression data using edgeR package and then projected the data into the lower dimensional space using multidimensional scaling approach (51). The data from the first 2 dimensions were used for the further investigation.

### Identification of common and disease-specific genes between distinct disease etiologies.

The pseudo-bulk gene count data for each cell type was used for this analysis, and only the datasets with at least 2 control samples were selected for further consideration. We focused on the disease states of IPF, ILD-not-IPF, COPD, fibrosis following COVID-19 infection, and CLAD, as these diseases had a sufficient sample size for the analysis. Ribosomal protein and mitochondrial genes were removed, keeping only protein-coding genes for the analysis. To avoid potential study bias, we selected genes detected in at least 1 sample from each study. One sample from the Morse et al. dataset (SC249NORbal) was removed because it used different reference genome patch release versions compared with the other samples in the same studies. Additionally, we excluded the Sun et al. dataset, as it used transplant rejection lungs as controls, conflicting with our study of CLAD. We used the edgeR package for differential gene expression and included only cell types with at least 2 disease states for further analysis. A linear model was fitted, and post hoc comparison analysis was subsequently performed to identify common and disease-specific genes. Common genes met the criteria of being significantly different from controls with an adjusted *P* value < 0.05 and a fold-change cutoff of 1.35, a percentage of sample expression above the cutoff (0.1 for upregulated genes and 0.6 for downregulated genes to avoid false positives), consistent regulation across all diseases, and significance in all 5 diseases. Disease-specific genes met the criteria of significant difference from controls with an adjusted *P* value < 0.05 and a fold change cutoff of 1.35, a percentage of sample expression above the cutoff, significantly higher expression compared with other disease states with a fold-change cutoff of 2, and median logCPM values (after batch correction) ranking highest compared with other diseases.

### PPI analysis.

The significantly upregulated DUGs for each disease and cell type were used for PPI analysis. The STRINGdb R package was employed to interface with the STRING database (Version 11.5), using a minimum confidence score of 400 for interactions (52). PPI networks were generated by mapping gene identifiers to corresponding STRING database IDs.

### Benchmarking pseudo-bulk versus FACS-sorted AT2 in IPF.

We downloaded the top 50 significant upregulated and downregulated genes for FACS-isolated AT2 cells (GEO GSE94555) from Cincinnati Children’s Hospital Medical Center Lung Gene Expression Analysis Web Portal (https://research.cchmc.org/pbge/lunggens/lungDisease/diffgene_IPF_type2.html?cid=up). Using our pseudo-bulk RNA-Seq data, we identified differentially expressed genes by comparing with controls, setting a significance threshold of FDR < 0.05. We then performed an overlay analysis to compare our identified genes with the reported top 50 significant genes.

### Obtaining the core gene signature of KRT17^+^KRT5^–^ cells.

To derive the core gene signature of KRT17^+^KRT5^–^ cells, we used pseudo-bulk gene count data for all epithelial cells, excluding PNEC_Ionocyte and Serous cells because of their small sample sizes. We retained only protein-coding genes, removing ribosomal protein and mitochondrial genes. We ensured each gene’s presence across all studies, keeping those detected in at least 1 sample per study. A linear additive model, incorporating cell type information and sample ID as covariates, accounted for technical effects and differences between disease etiologies. Differential gene expression analysis between KRT17^+^KRT5^–^ cells and other epithelial cells (AT1, AT2, Club, Ciliated, Differentiating Ciliated, Goblet, and Basal cells) was performed. The core KRT17^+^KRT5^–^ cells gene signature was defined using the following criteria: 1) significant difference compared with the average of other epithelial cells with an FDR less than 0.05, 2) a fold change cutoff of at least 2, and 3) a higher median logCPM value in KRT17^+^KRT5^–^ cells compared with other epithelial cell types. For the pathway identification, we used the Hallmark and Reactome databases from Molecular Signatures Database repository as the reference. The pathways with *q* value less than 0.05 were regarded as the significant pathways.

### Cell interactome analysis of KRT17^+^KRT5^–^ cells using pseudo-bulk RNA-Seq data.

We used batch-corrected count data from diseased lungs for this analysis, excluding the SC249NORbal sample from the Morse et al. dataset due to a different reference genome version. The diseases analyzed included IPF, ILD-not-IPF, and COVID-19, with sample sizes of 17, 5, and 25, respectively. CLAD and COPD were excluded because of small/no sample size. We used the CellChat program, excluding interacting cell types if the patient number was less than 3. The communication probability between interacting cell groups was computed using the “trimean” method, selecting significant signaling and ligand-receptor pairs. Only pathways involving the KRT17^+^KRT5^–^ core gene signature were selected for further analysis.

### Bioinformatics analysis of Xenium data of IPF lungs.

The image-based Xenium experiment was performed at the Metabolomics Core Facility at NU, following the product protocols. The Seurat package was used to analyze the Xenium output data, and cells with fewer than 3 molecular counts were excluded from further analysis. In addition to the 289 predesigned lung genes, we designed an additional 100 genes to facilitate our study. Cell type annotation was based on the cell type gene markers, and subsequent bioinformatics analysis followed the Seurat package pipeline.

### Single-nucleus and single-cell RNA-Seq of COVID-19/CLAD samples and bioinformatics analysis.

To investigate snRNA-Seq in COVID-19, we obtained lung tissue from the explanted lungs of 3 patients with long-term COVID-19 who experienced fibroproliferative acute respiratory distress syndrome and received double lung transplants. The tissue was promptly frozen in liquid nitrogen and stored at –80°C until processing, following a previously published protocol for single-nucleus isolation (53). For the scRNA-Seq study of CLAD, we used healthy donor lungs not designated for transplantation as controls, selecting 4 CLAD and 4 control samples. The Chromium Single Cell V3 Reagent Kit (10x Genomics) was used for library preparation. Library quality was assessed using TapeStation 4200 (Agilent), followed by sequencing on a HiSeq 4000 instrument (Illumina). Sequencing reads for COVID-19 data were aligned to the human genome HG38, then augmented with the entire SARS-CoV-2 genome (RefSeq assembly accession: GCF_009858895.2), using the Cell Ranger version 6.0 pipeline (10x Genomics). Both scRNA-Seq and snRNA-Seq data underwent removal of ambient RNAs using the SoupX toolkit, and doublets were identified and removed using the scDblFinder package. Further quality control measures were applied using the Seurat package, removing cells with extreme feature counts or RNA counts, or high mitochondrial percentages. Subclustering analysis and differential gene expression analysis were conducted using Seurat package and the Harmony approach. Enrichment scores were calculated using the “AddModuleScore” function in Seurat.

### Donor-recipient cell deconvolution in CLAD lungs.

We used Cellsnp-lite, a highly efficient software for genotyping in single-cell sequencing data, to identify genetic variants within each individual cell (54). The possorted_genome_bam.bam file generated by Cell Ranger served as the input for Cellsnp-lite. A quality list of candidate SNPs with allele frequency > 5% (downloaded from https://sourceforge.net/projects/cellsnp/files/SNPlist) was used to pile up the genetic variants. For filtering, we excluded variants with < 20 UMIs or < 10% minor alleles, as recommended by Cellsnp-lite. The filtered genotypes were then processed using the Vireo package for donor and recipient cell demultiplexing with default parameters. Cells with a probability < 0.9 of being assigned to a specific group were excluded from further analysis, as were doublets predicted by Vireo. Additionally, we used cell type annotations to classify cells: Those predominantly composed of structural cell types were categorized as donor cells, while cells dominated by immune cells were classified as recipient cells. To determine the proportion of recipient cells for each cell type, we calculated the ratio of recipient cells to the total cell count for each patient. In cases where the total cell count was fewer than 20, we manually designated the proportion as “not applicable.”

### Statistics.

For comparisons between 2 groups, the Wilcoxon test was used. For comparisons involving more than 2 groups, the Kruskal-Wallis test was followed by pairwise Wilcoxon rank-sum tests. *P* values were adjusted using the BH method, and FDR < 0.05 was considered statistically significant.

### Study approval.

This study was approved by the IRB at Northwestern University.

### Data availability.

The single-cell transcriptomic data of CLAD scRNA-Seq have been deposited in the GEO under accession number GSE289881. Source code and documentations are freely available at https://github.com/yuanqingyan/singleGEO; commit ID cb24839.

## Author contributions

YY and AB contributed to conceptualization and study design. YY, RG, GRSB, and AB contributed to formal analysis, methodology, and data interpretation. EL, XW, MS, HS, and FLNS conducted experiments. TK, CK, and AB contributed to sample collection.

## Funding support

This work is the result of NIH funding, in whole or in part, and is subject to the NIH Public Access Policy. Through acceptance of this federal funding, the NIH has been given a right to make the work publicly available in PubMed Central.

NIH HL145478, HL147290, HL147575, HL173940, P01HL169188 (to AB).

NIH P01 AG049665, P01 HL071643 (to GRSB).

NIH 1R01HL160552, R35GM142539 (to RG).

## Supplementary Material

Supplemental data

Supporting data values

## Version 1 10/22/2025

Electronic publication

## Version 2 10/29/2025

Figures 5 and 7 were replaced: corrections were made for clarity
