# Supplementary material for: Single-cell dissection of chronic lung allograft dysfunction reveals convergent and distinct fibrotic mechanisms
Source: JCI Insight. 2025 Oct 22;10(20):e197579. doi: 10.1172/jci.insight.197579 (PMC12581678; doi:10.1172/jci.insight.197579)
Supplement: Supplemental data [file jciinsight-10-197579-s281.pdf]

Supplemental Figures

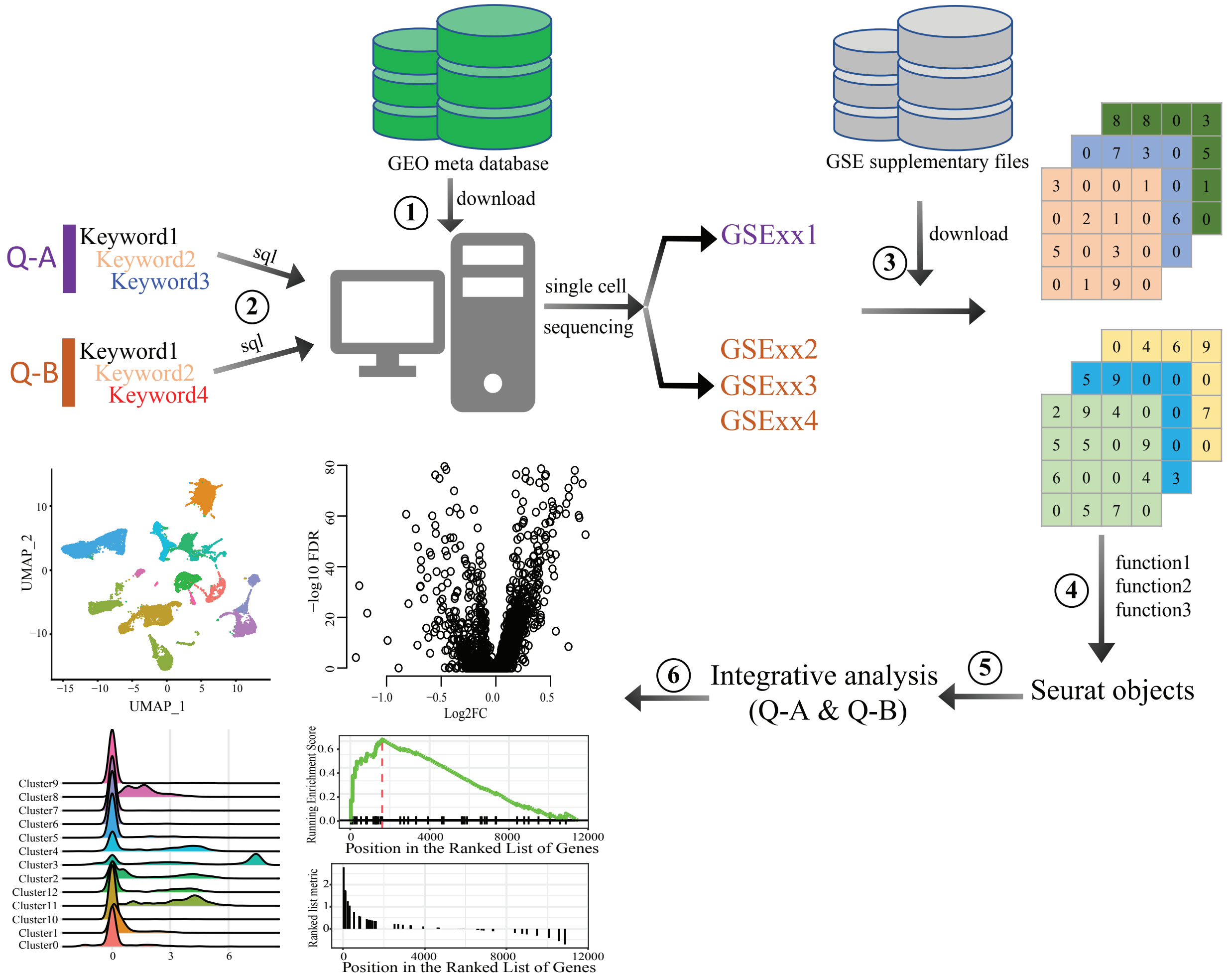

Figure S1. Schematic diagram of the SingleGEO development pipeline. SingleGEO streamlines the systematic identification of single-cell sequencing datasets by interacting with the GEO database, eliminating the need for manual dataset searches. The package consists of multiple steps, including GEO meta-database downloading, keyword-based querying, targeted dataset selection, single-cell data retrieval, data quality control, and Seurat object construction for integrative analysis. First, the GEO meta-database is downloaded locally, followed by the implementation of a fast, lightweight search engine to query the database based on user-defined keywords. The search returns relevant metadata for further examination. Once specific GSE IDs are selected, the corresponding single-cell sequencing data from GEO supplements can be downloaded for downstream analysis. Using the Seurat package, data quality control and

integrative analysis can then be performed. Detailed usage instructions are available at https://github.com/yuanqingyan/singleGEO/vignettes.

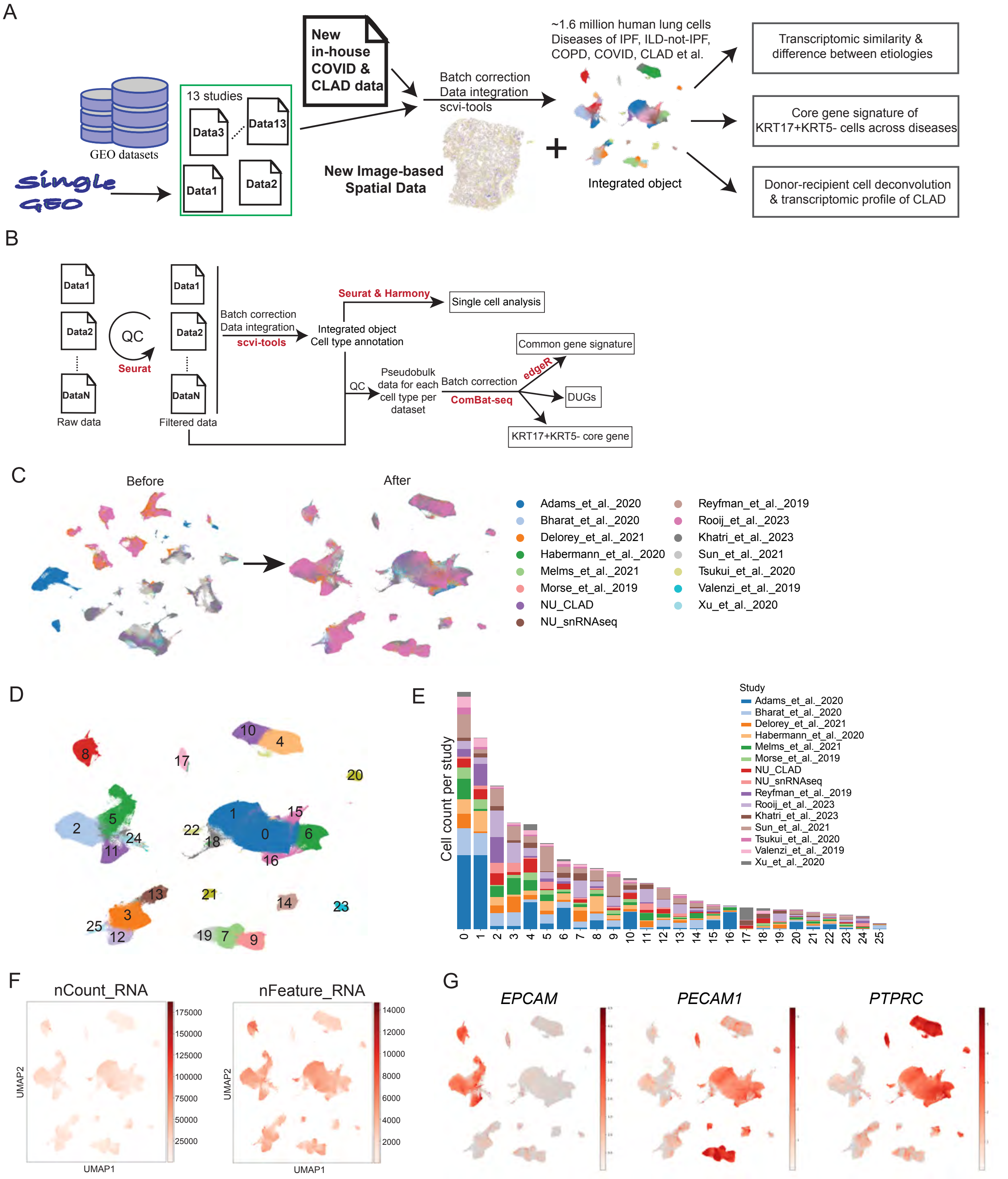

Figure S2. Quality control of data integration using scvi-tools. (A) The schematic diagram of the bioinformatics analysis in this study, including database query, new data generation, data quality control, data integration and the subsequent analysis. (B) Schematic illustrating the different toolkits used in this study. (C) UMAP plot showing cell clustering before and after data integration with scvi-tools. (D) Leiden clustering results derived from the batch effect-corrected dataset. (E) Distribution of studies within each identified cell cluster. (F) Distribution of gene count

and UMI count across cell clusters. (G) The identification of four cellular compartments based on gene expression levels of *EPCAM, PECAM1,* and *PTPRC*.

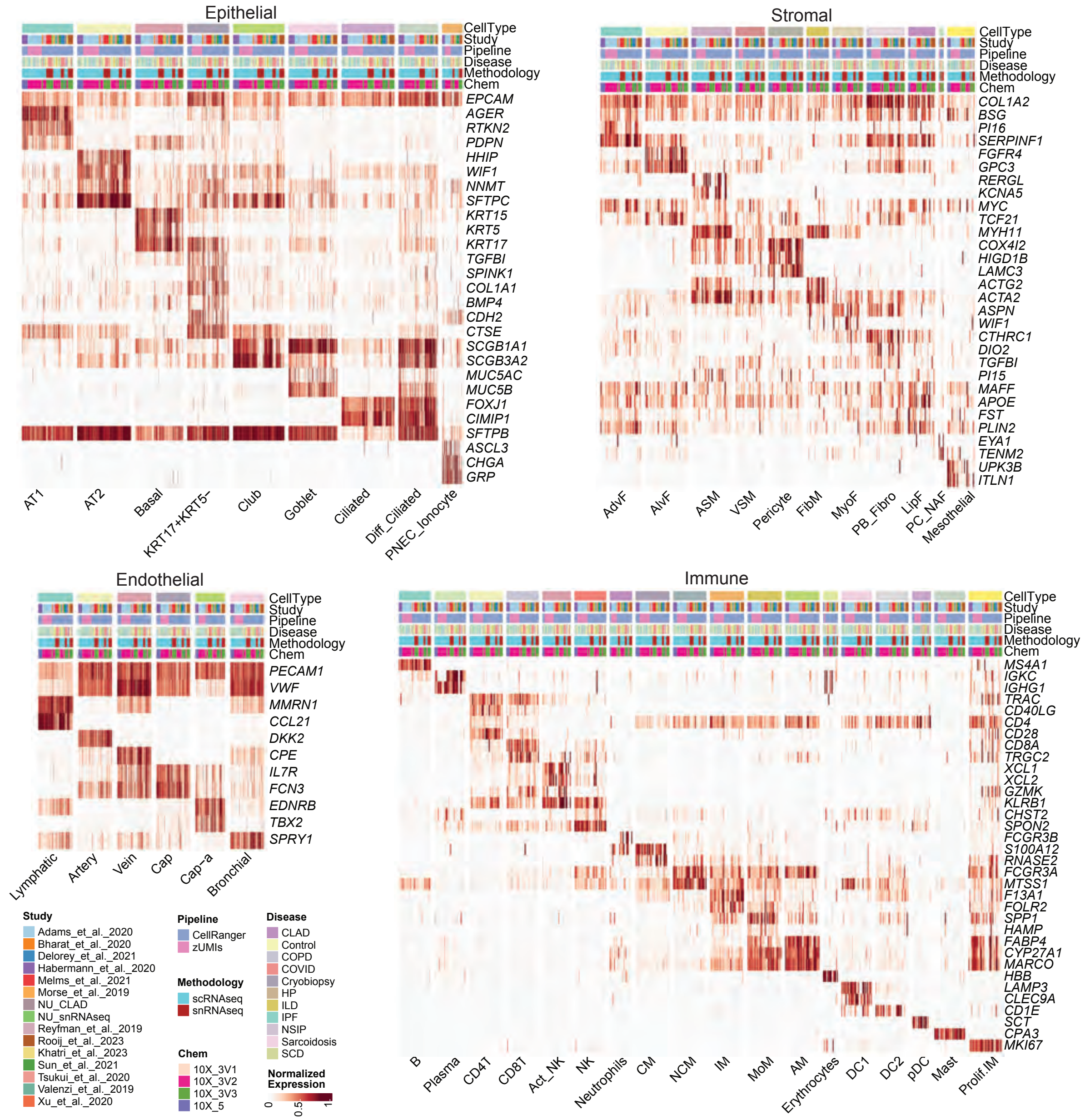

Figure S3. Heatmap of marker gene expression for each cell type at the patient level. Each column represents an individual subject. The averaged expression level for each gene per cell type was calculated from the normalized expression data for each individual. These averaged values were then normalized within each dataset to the same range (0 to 1) to account for dataset-specific effects.

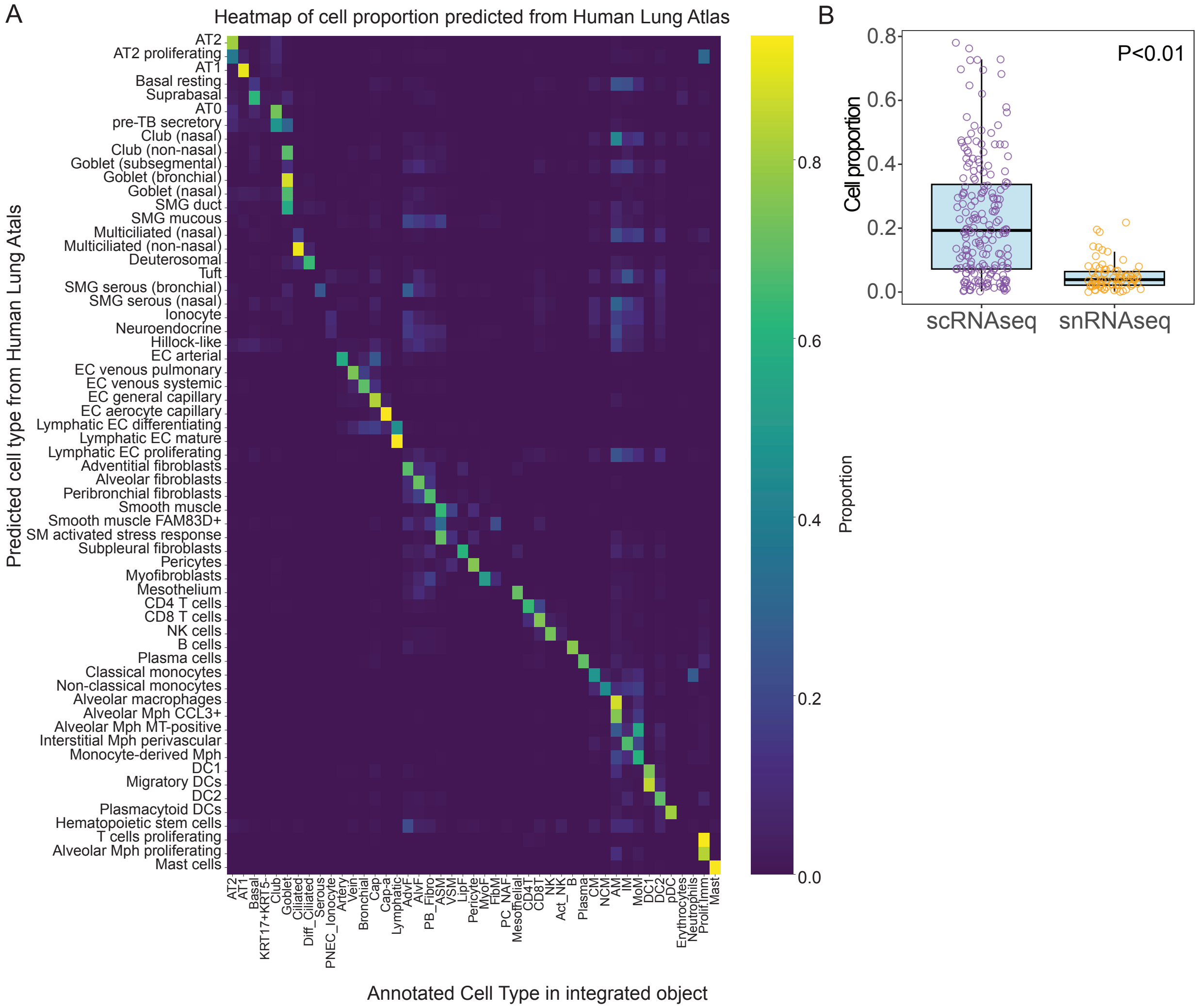

Figure S4. Concordance of cell type annotations with the Human Lung Atlas. (A) The CellTypist program was used to predict cell types using the Human_Lung_Atlas model. The proportion of predicted cell types for each annotated cell type was calculated, with higher proportions indicating greater concordance between our annotated dataset and the Human Lung Atlas annotations. (B) Boxplot to illustrate the cell proportion different of AM between scRNAseq and snRNAseq. The cell proportion was calculated by the count of AM normalized to the total cell number per sample. Wilcoxon test was used to evaluate the statistical significance.

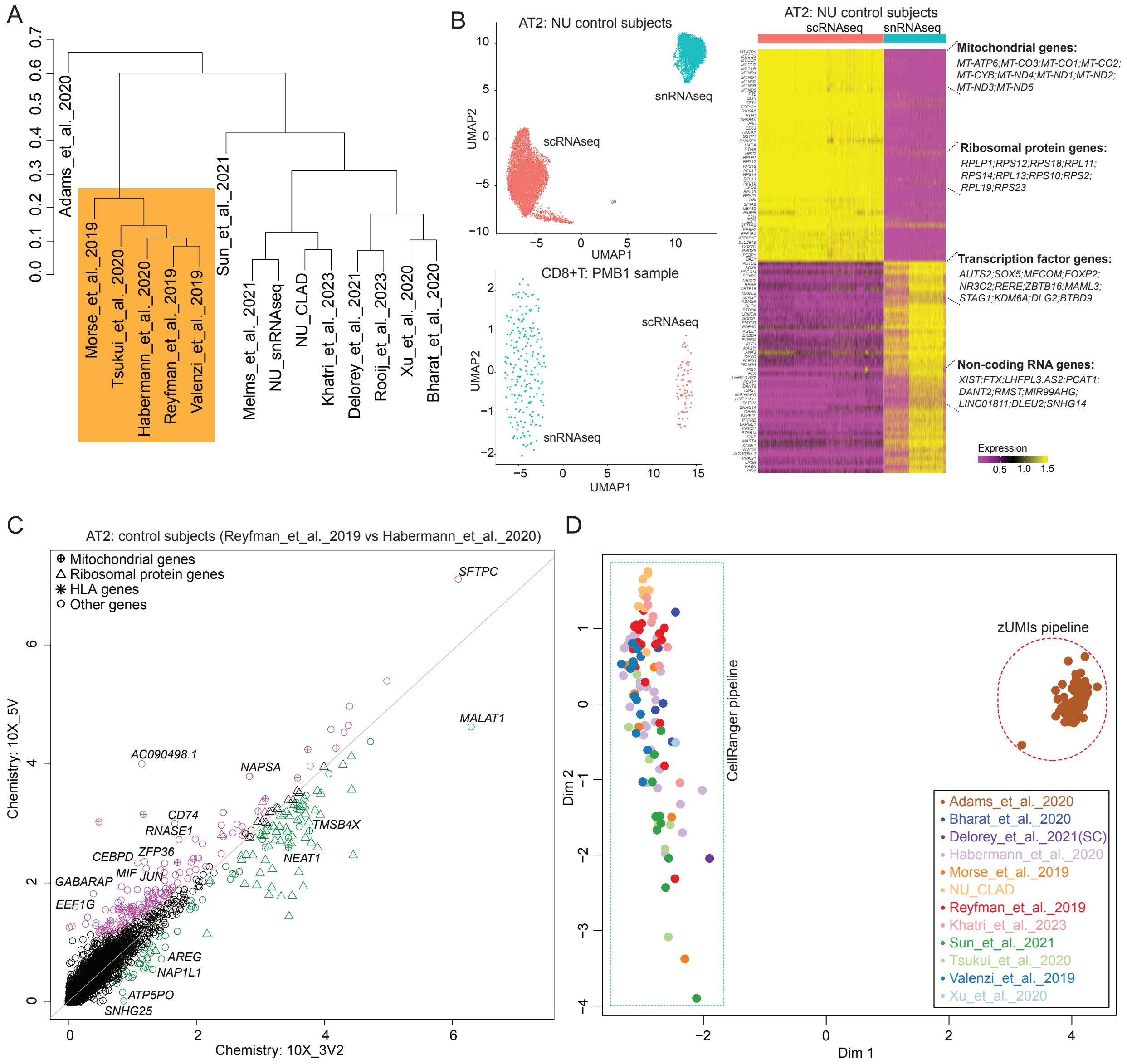

Figure S5. Four types of technical factors impending cross-study single-cell transcriptomic analysis. (A) The biases introduced by utilizing different reference genome builds are exemplified by hierarchical clustering of studies based on gene presence, which reveals that studies employing an outdated reference genome patch release version tend to cluster together. (B) The biases introduced by different library preparation methods for transcriptomic profiling are exemplified by the case of AT2 cells. The UMAP visualization of AT2 sub-clusters was derived from control samples of 3 NU studies (upper left) and a COVID-19 sample (PMB1) prepared with both scRNAseq and snRNAseq (bottom left). The right panel shows the heatmap of selected genes from AT2 of control samples from 3 NU studies. (C) The scatter plot of mean gene expression levels in AT2 control samples from the Reyfman et al. study (using 10X 3V2 chemistry) and the Habermann et al. study (using 10X V5 chemistry) illustrates the biases introduced by different library preparation chemistries in transcriptomic profiling. (D) The biases introduced by different data processing pipelines are exemplified by the MDS plot of pseudo-bulk RNA samples, which shows distinct clustering of samples from different studies. The pseudo-bulk count was

obtained from alveolar macrophage and only the datasets sequenced by scRNAseq method were used.

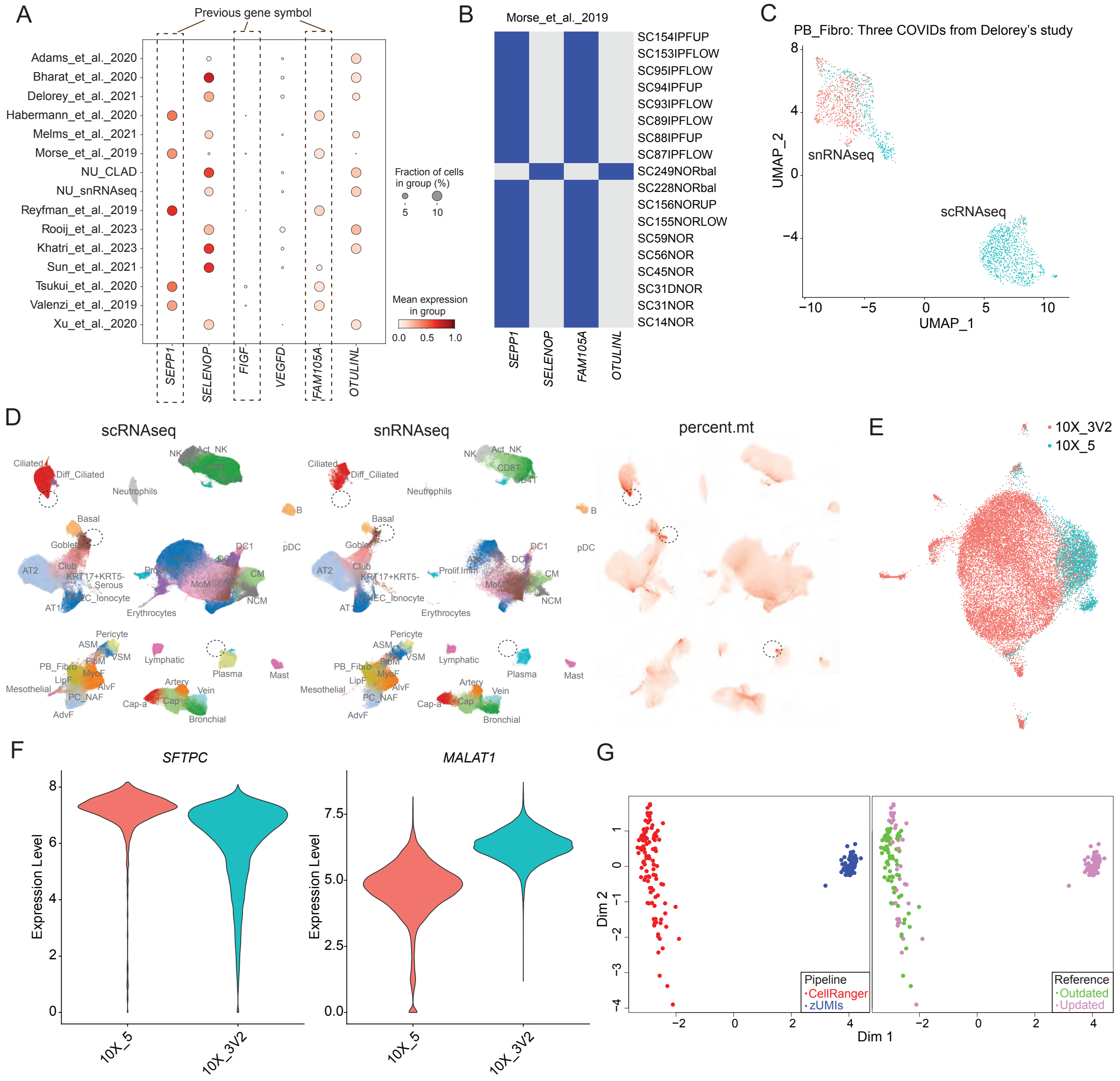

Figure S6. Detailed technical effects within the single cell integrated object. (A) Dot plots of the expression of three pairs of updated and outdated genes (*SELENOP* vs *SEPP1*; *VDGFD* vs *FIGF*; *OTULINL* vs *FAM105A*) within each study. (B) Heatmap of gene expression for updated and outdated gene symbols in each sample from the Morse et al. dataset. (C) Sub-clustering analysis of PB_Fibro from three COVID-19 samples, prepared using both scRNAseq and snRNAseq from the Delorey et al. study. (D) UMAP visualization showing the presence of cellular subsets with high levels of mitochondrial genes in both scRNAseq and snRNAseq datasets. (E) Sub-clustering analysis of AT2 cells from the Reyfman et al. (10X 3V2) and Habermann et al. (10X V5) studies. (F) Violin plots of *SFTPC* and *MALAT1* gene expression between two different single-cell chemistries (10X 3V2 and 10X 5V). (G) MDS plot illustrating the sample distribution, colored by different features.

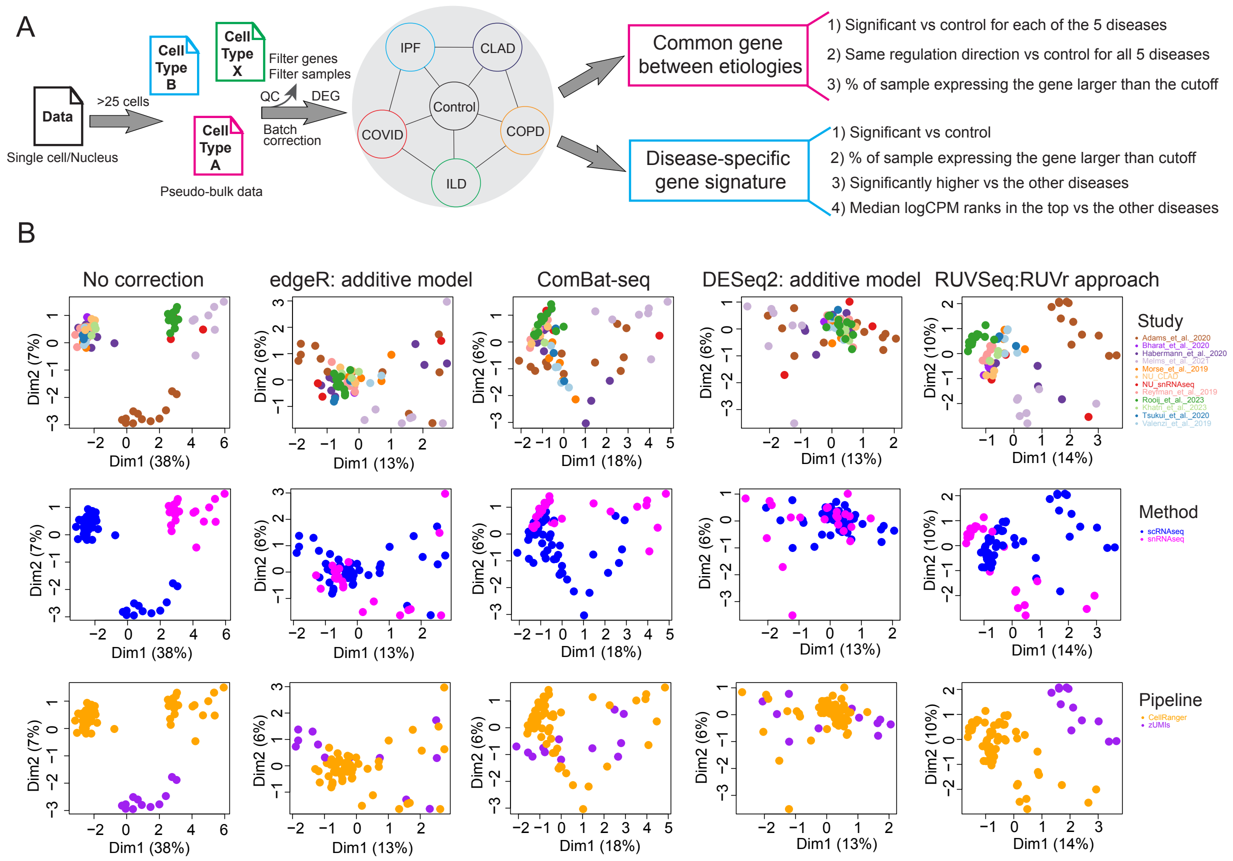

Figure S7. MDS plot showing sample distribution to benchmark different programs for batch effect correction. (A) Schematic of the analysis approach using pseudo-bulk RNA sequencing, including calculation of cell type-specific counts, data quality control, batch effect correction, differential gene expression analysis, and criteria for selecting gene signatures. (B) MDS plot of benchmarking results. The control samples from the AT2 cell type were used in this analysis. Samples are colored by study (upper panel), library preparation method (middle panel), and computational pipeline (lower panel).

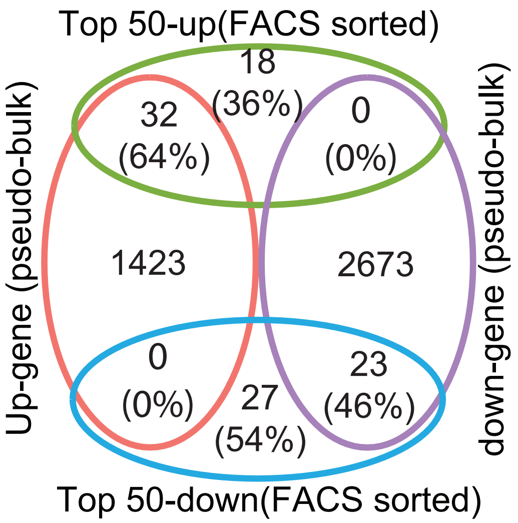

Figure S8. Venn diagram to illustrate the overlay of significant genes between our pseudo-bulk analysis and FACS-sorted AT2 cells. By talking the top 50 up-regulated genes in AT2 data from FACS-sorted as ground truth, our pseudo-bulk RNAseq approach correctly identified 32 (64%) of them and none of these was misanalysed as down-regulated genes. By comparing the top 50 down-regulated IPF genes in FACS-isolated AT2, 23 (46%) of them showing significantly down-regulated in our analysis and none of them was misanalysed as up-regulated genes

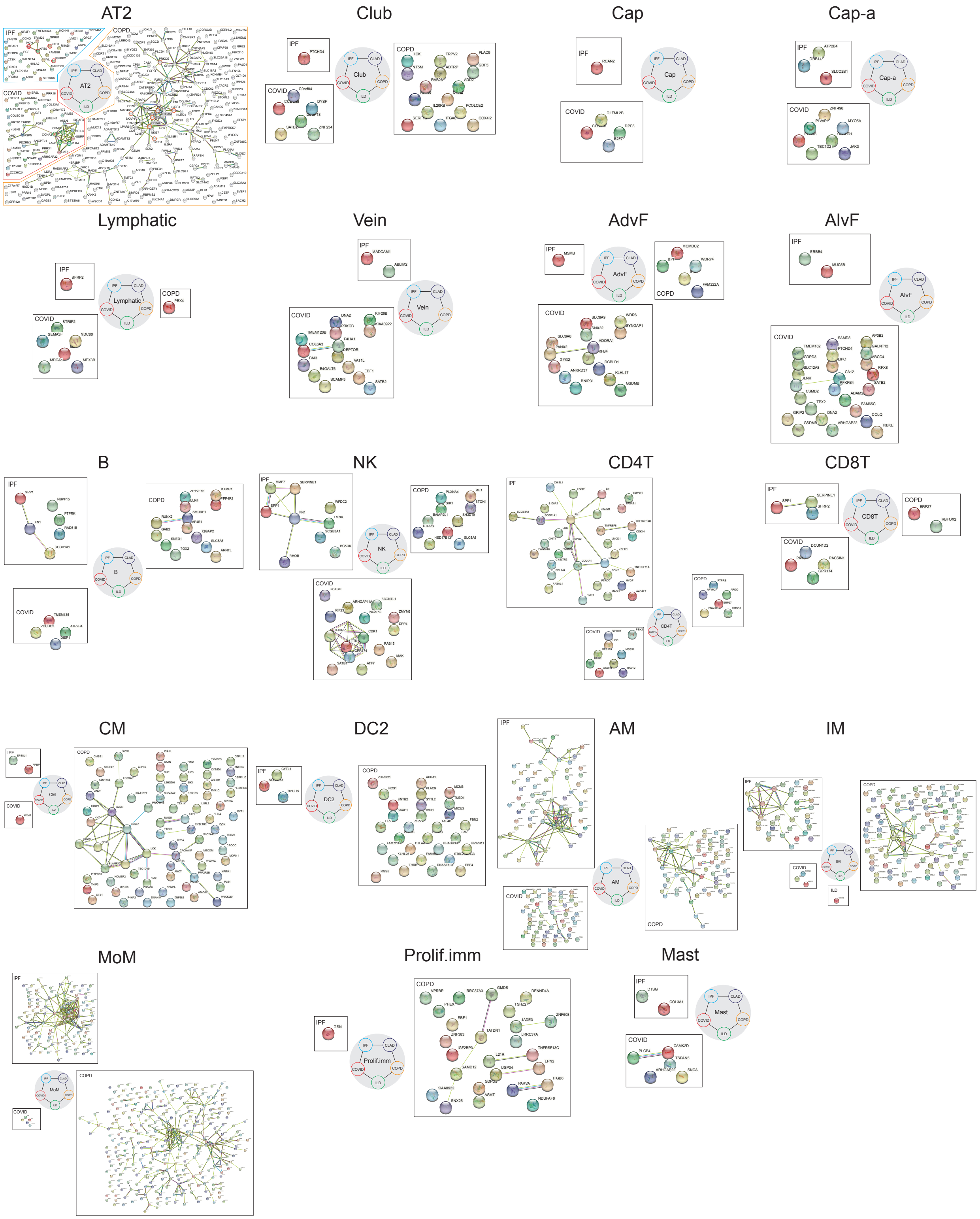

Figure S9. Protein-protein interaction network of DUGs in different cell type for different diseases.

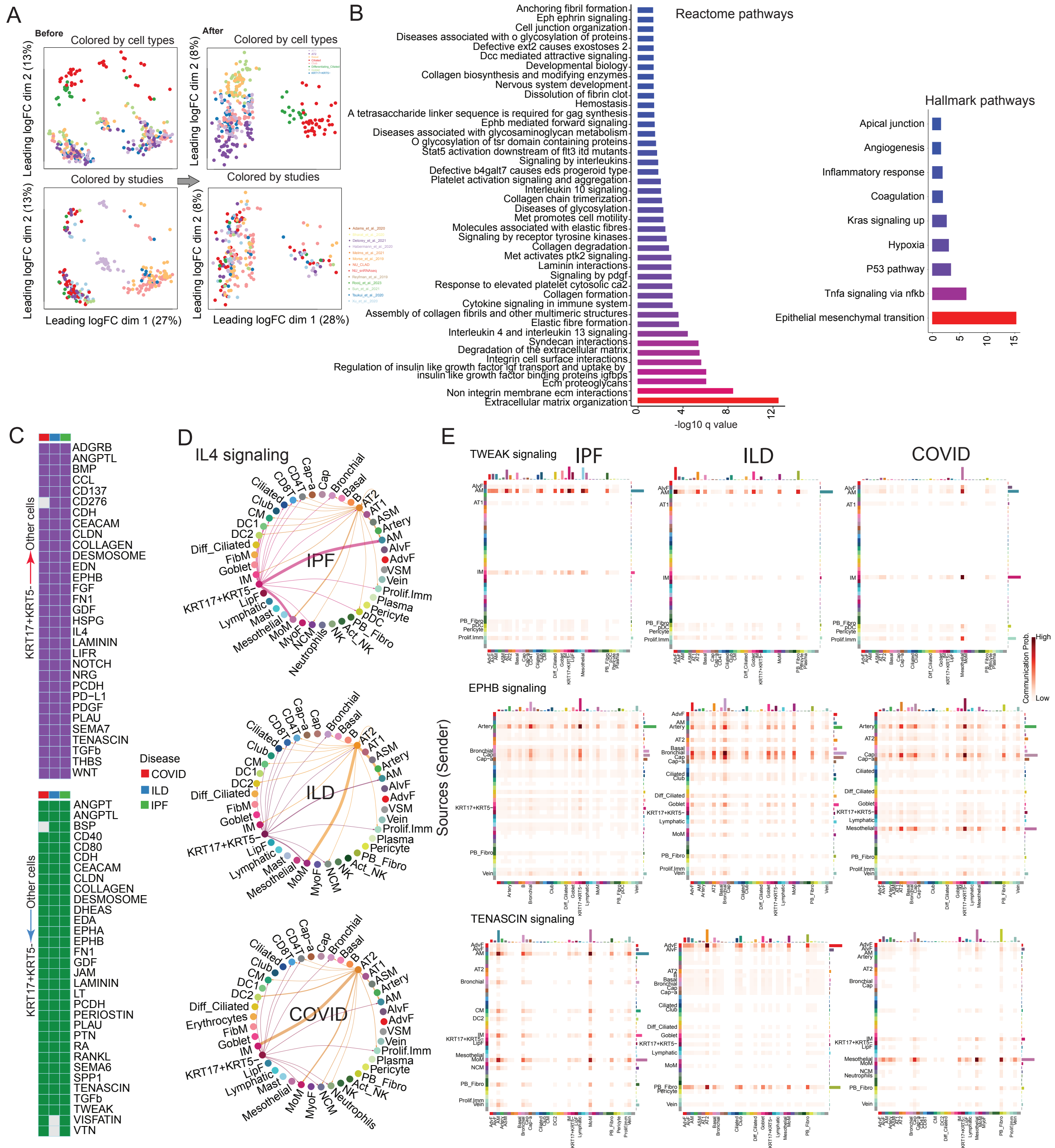

Figure S10. Transcriptomic profile of KRT17+KRT5- cells in different diseases. (A) MDS plots of samples showing the success of batch effect correction to obtain core KRT17+KRT5- gene signature. (B) The significant Hallmark and Reactome pathways enriched by the 360 core genes. (C) Heatmap showing significant signaling pathways between KRT17+KRT5- cells and other cell types, analyzing only pathways involving core genes. (D) Circle plot illustrating the interaction network of IL4 signaling between KRT17+KRT5- cells and other cell types. (E) Heatmap depicting the signal flow of TWEAK, EPHB and TENASCIN signaling among various cell types across distinct disease conditions.

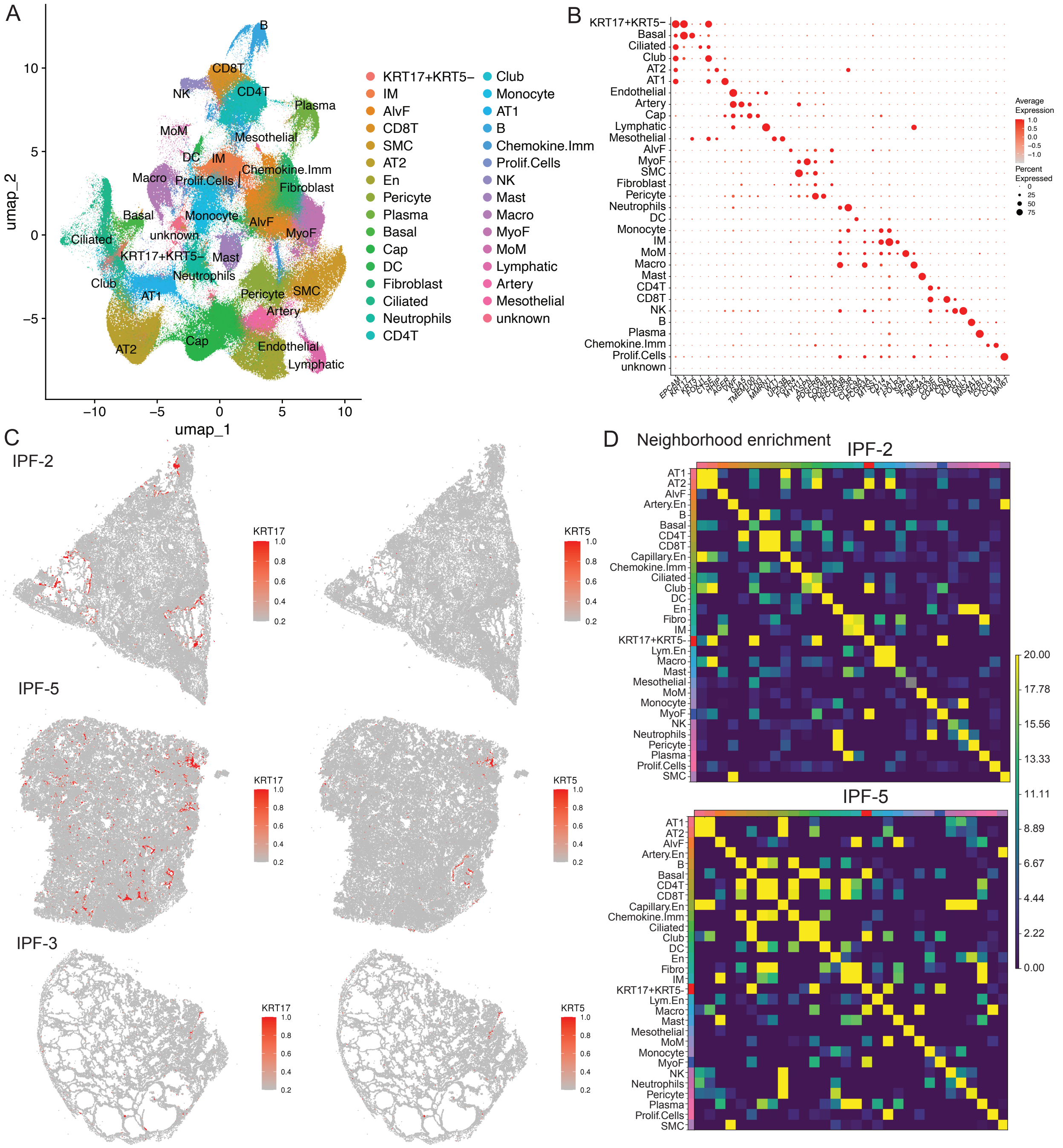

Figure S11. Cell type annotation of gene expression of image-based spatial transcriptome of IPF lungs. (A) UMAP visualization of different cell types identified from image-based spatial Xenium platform. (B) Dot plot of expression levels of cell type-specific gene markers. (C) Expression of *KRT17* and *KRT5* in IPF lungs in the spatial context. (D) Heatmap depicting neighborhood enrichment analysis, illustrating the interactions between KRT17+KRT5- cells and other cell types within the fibrotic regions of IPF lungs.

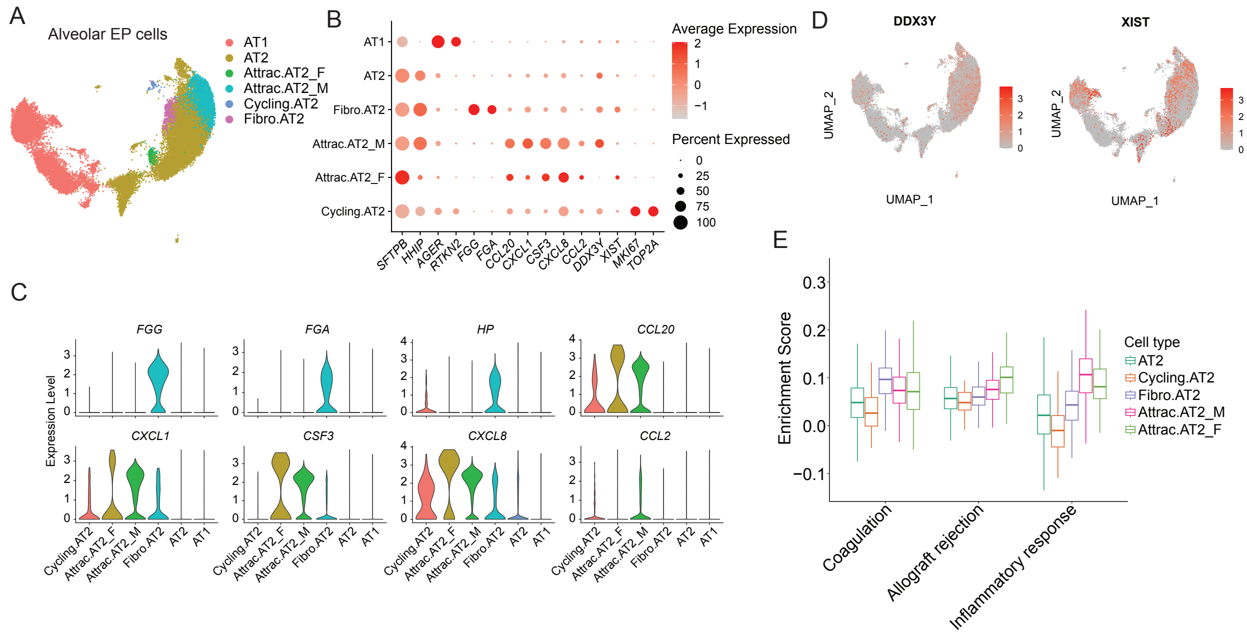

Figure S12. Differential cellular and molecular signatures of epithelial cells in CLAD. (A) UMAP visualization of alveolar epithelial cell subsets. (B) Dot plot displaying gene expression levels across different cell subsets, with z-score transformed values. (C) Violin plots highlighting key genes distinguishing each subset. (D) Feature plots of two genes differentiating the Attract.AT2_M and Attract.AT2_F subsets. (E) Boxplot showing differential enrichment scores of three pathways among cell subtypes.

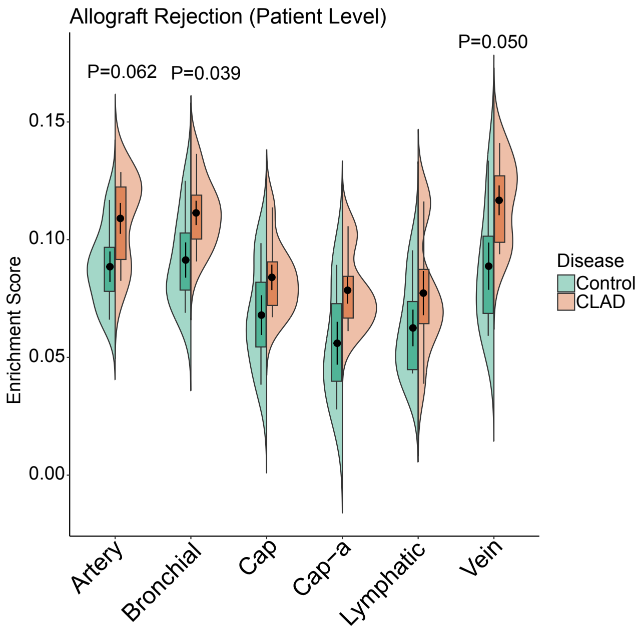

Figure S13. Differential gene expression between donor and recipient cells in endothelial cells. Violin plots illustrate the allograft rejection pathway enrichment scores in endothelial cells, comparing CLAD and control samples. Statistical significance was determined using a Student's t-test.

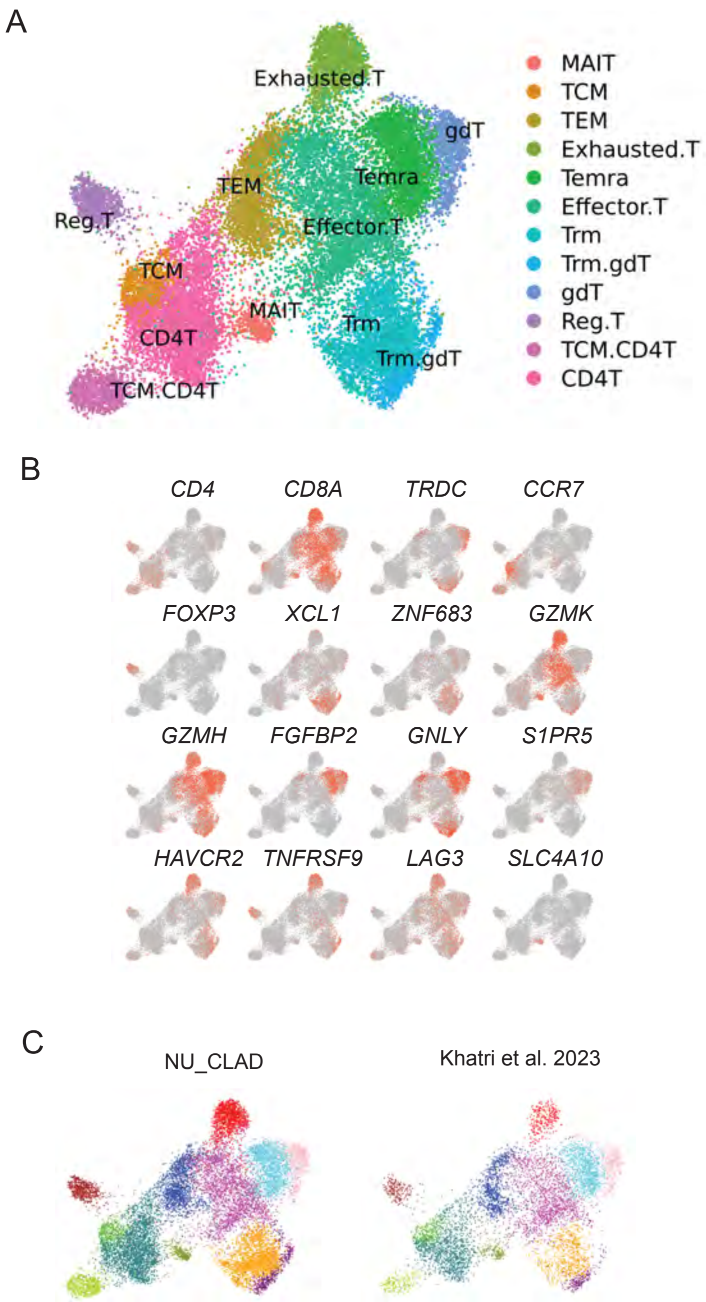

Figure S14. Unique cellular and molecular profiles of lymphoid cells in CLAD. (A) UMAP visualization of different T cell subtypes within the T cell lineage. (B) Feature plots showing marker gene expression across different T cell subtypes. (C) UMAP visualization of T cell lineage distribution across different studies.

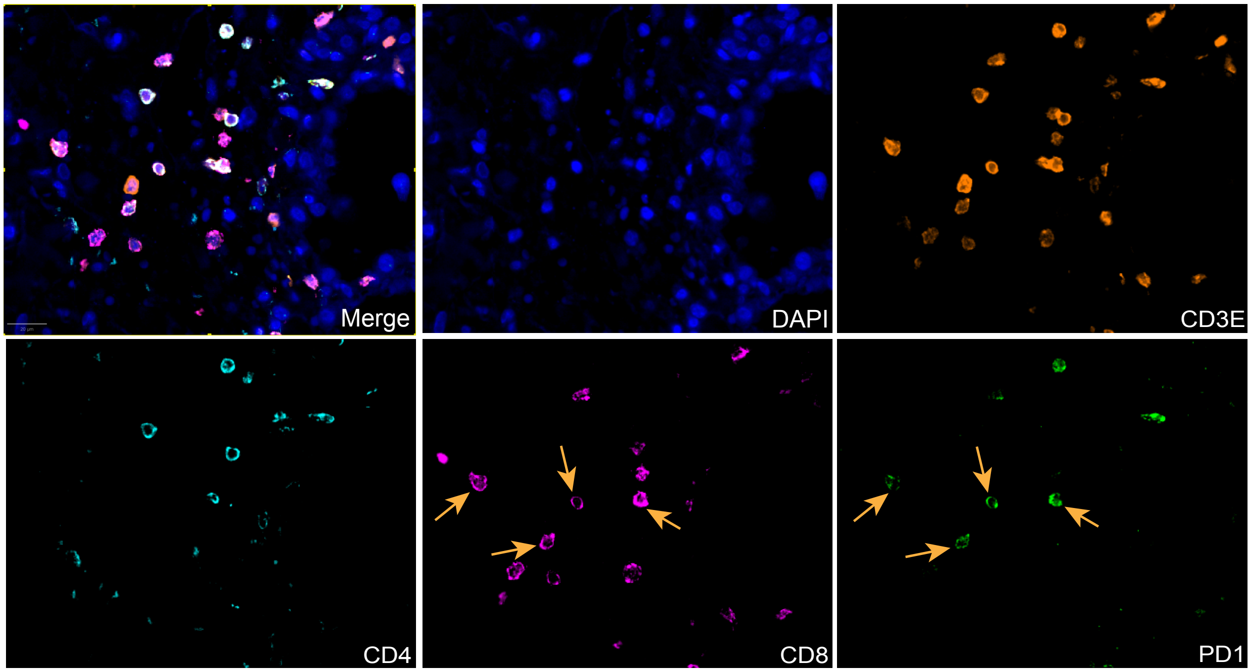

Figure S15. Immunofluorescence staining of Exhausted.T in CLAD lungs. These cells were identified by the overexpression of CD3E, CD8, and PD1 markers. Arrows indicate the presence of Exhausted.T cells.

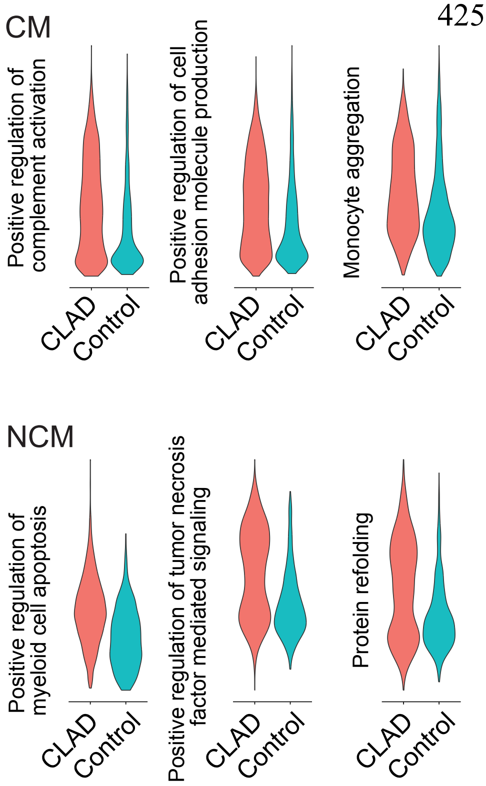

Figure S16. Differential cellular and molecular profiles of myeloid cells in CLAD. Violin plot comparing differential enrichment scores for biological processes between CLAD and controls in CM and NCM.

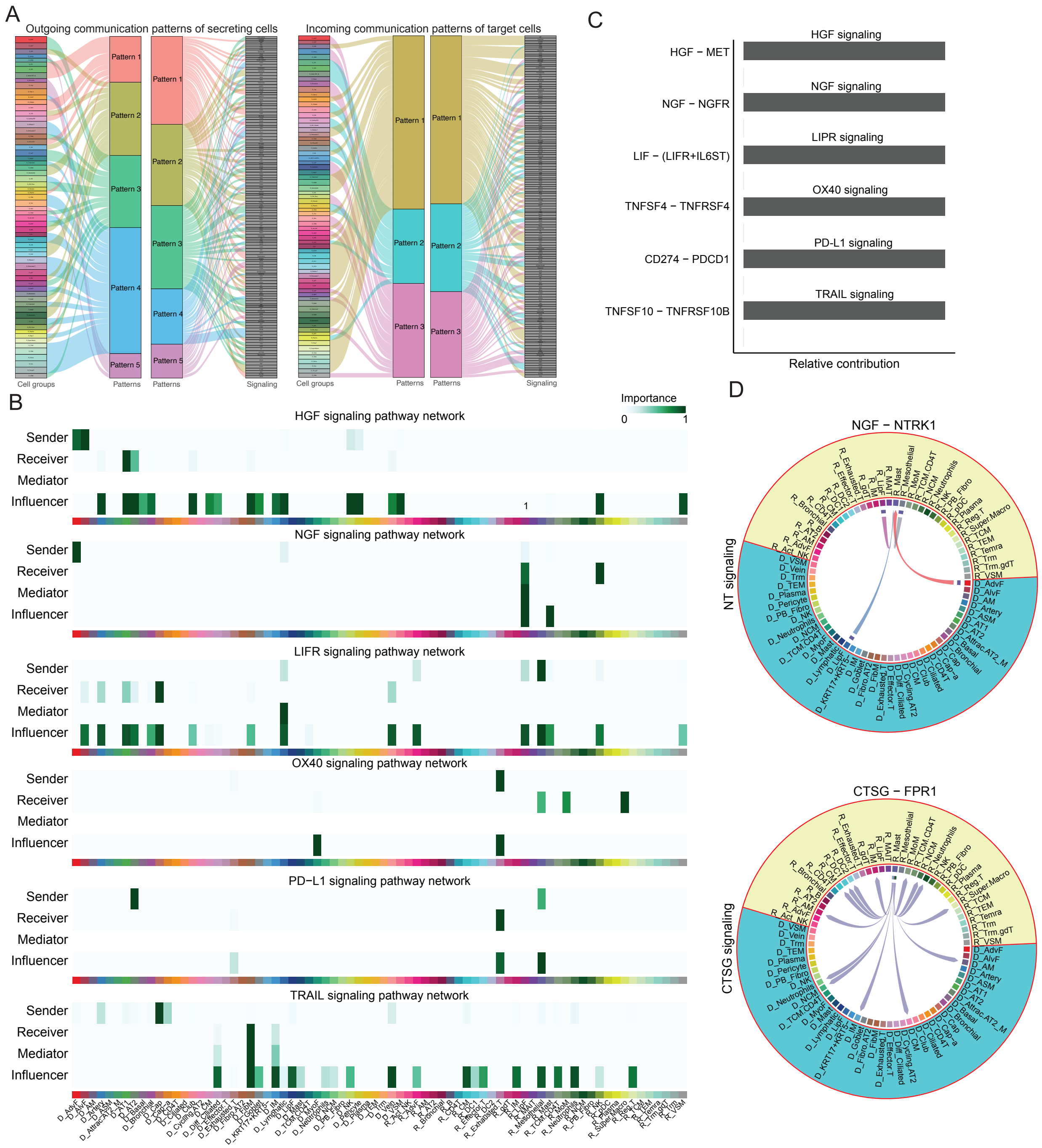

Figure S17. Cell-cell communication analysis in the CLAD microenvironment. (A) The different outgoing communication patterns of secreted cells and incoming communication patterns of target cells. (B) Heatmap showing the involvement of different cell types in six signaling pathways inferred from cell-cell interaction analysis. (C) The ligand-receptor pairs involved in the six signaling pathways. (D) Circle plots illustrating signal flow in two signaling pathways.
